# The PLAU-ITGB1-TGF-β axis orchestrates spatiotemporal reprogramming of fibroblasts at the invasive front to drive dual immunosuppressive niche and immunotherapy resistance in oral squamous cell carcinoma

**DOI:** 10.1038/s41419-026-09110-4

**Published:** 2026-07-16

**Authors:** Yili Xiong, Shaopeng Liu, Lianyu Zhao, Xinze Liu, Hanzhi Zhang, Haoran Wang, Dongsheng Zhang, Changlong Wang, Haiwei Wu

**Affiliations:** 1 Department of Stomatology, Shandong Provincial Hospital Affiliated to Shandong First Medical University, Jinan, Shandong 250021 P.R. China; 2 School of Stomatology, Shandong First Medical University & Shandong Academy of Medical Sciences, Jinan, Shandong 250117 P.R. China; 3 School of Life Sciences, Shandong First Medical University & Shandong Academy of Medical Sciences, Tai’an, Shandong 271016 P.R. China; 4 Medical Science and Technology Innovation Center, Shandong First Medical University & Shandong Academy of Medical Sciences, Jinan, Shandong 250117 P.R. China

**Keywords:** Oral cancer, Mechanisms of disease, Immune evasion, Cell signalling, Next-generation sequencing

## Abstract

The initiation and progression of cancer are driven by the dynamic interplay between somatic mutations and the tumor microenvironment. Identifying core cell populations driving malignant transformation and understanding the intercellular interactions within the tumor microenvironment are, therefore, crucial for early diagnosis and effective treatment. Here, we construct a spatiotemporal atlas of oral squamous cell carcinoma (OSCC) progression by integrating multi-omics approaches. Our analysis reveals that the critical driver gene PLAU activates the TGF-β pathway via ITGB1, thereby promoting the conversion of fibroblasts into the COL11A1⁺ fibroblast (COL11A1⁺ Fib). These specialized fibroblasts remodel the extracellular matrix (ECM), collectively establishing an immunosuppressive niche composed of malignant epithelial cells, COL11A1^+^ Fib, and regulatory T cells (Tregs). Importantly, targeting COL11A1^+^ Fib alleviates this immunosuppressive niche and curbs OSCC progression. Our findings underscore the potential of COL11A1^+^ Fib as a predictive biomarker, especially in combination with immunotherapy. Collectively, this work demonstrates that the PLAU-ITGB1-TGF-β axis drives the formation of an Epi-COL11A1^+^ Fib-Treg immunosuppressive niche that fuels OSCC malignancy.

**Figure:**
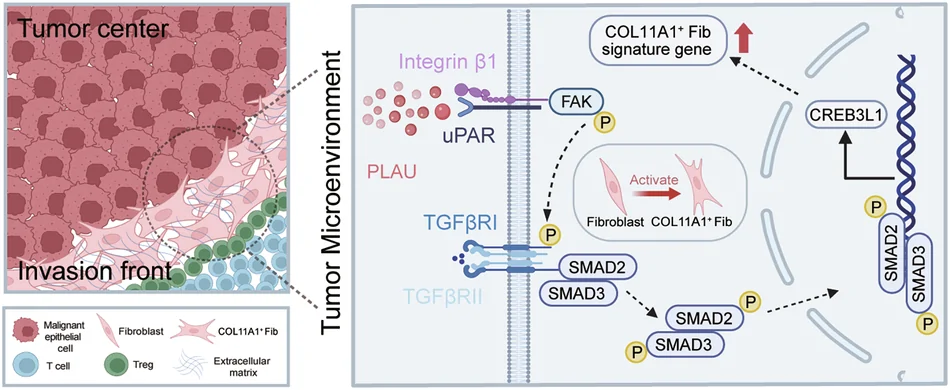

## Introduction

OSCC develops from normal oral mucosa to precancerous lesions and ultimately to invasive carcinoma [1]. Oral leukoplakia (OLK) is a representative precancerous lesion (Pre) with a risk of malignant transformation into OSCC of up to 20% [2]. Although the phenotypic and histopathological changes of malignant transformation are well established, how precancerous lesions progress to OSCC and the underlying molecular mechanisms remain incompletely understood.

Accumulating evidence indicates that OSCC development and progression are shaped by the diverse cellular constituents and their intricate interactions within the stromal microenvironment [3]. Fibroblasts play a critical role in the stromal microenvironment [4]. Recent single-cell RNA sequencing (scRNA-seq) analyses have revealed substantial fibroblast heterogeneity in OSCC, delineating distinct functional subpopulations. Moreover, certain fibroblast subtypes are associated with poor prognosis and resistance to immunotherapy [5, 6].

The stromal microenvironment shapes fibroblasts. Cancer cells can “educate” quiescent fibroblasts by secreting fibroblast growth factor (FGF), epidermal growth factor (EGF), and various inflammatory chemokines such as interleukin-6 (IL-6), thereby facilitating cancer expansion and metastasis [7–9]. Additionally, cancer cells can activate fibroblasts in situ through direct epithelial-stromal interactions via ANXA1-FPR2 signaling [10]. Besides cytokines, other environmental stressors may also be implicated in this process, possibly dependent on cancer progression stage [11]. Therefore, it is crucial to address how fibroblasts in the microenvironment are activated and how their activation leads to functional remodeling.

The urokinase-type plasminogen activator (PLAU) system, comprising PLAU, its receptor urokinase plasminogen activator receptor (uPAR), and its inhibitor plasminogen activator inhibitor-1 (PAI-1), is overexpressed in many tumors and plays a critical role in tumor development and metastasis [12, 13]. PLAU signals through uPAR; however, uPAR lacks intrinsic signaling capacity and requires co-receptors such as integrin β1 (ITGB1) [14]. Integrins mediate extracellular matrix (ECM) remodeling and promote cancer-associated fibroblast (CAF) deposition, thereby contributing to an immunosuppressive tumor microenvironment [15]. Collagen type XI alpha 1 (COL11A1), a component of type XI collagen, modulates tumor-stroma interactions and ECM mechanical properties, and promotes cancer cell migration [16].

In this study, we integrated multi-omics data with in vitro and in vivo experiments to identify and characterize the COL11A1^+^ fibroblast subpopulation (COL11A1^+^ Fib) in OSCC. Malignant epithelial cells secrete PLAU, PLAU acts through uPAR on fibroblasts to promote uPAR-ITGB1 interaction, leading to ITGB1 activation and subsequent phosphorylation of focal adhesion kinase (FAK). Activated FAK enhances the binding of TGFβRI to TGFβRII, thereby activating downstream Smad-dependent pathways. This signaling cascade also leads to CREB3L1 activation, driving transformation of fibroblasts into COL11A1^+^ Fib. These COL11A1^+^ Fib remodel the ECM and promote T cell differentiation, establishing a dual immunosuppressive niche at the invasive front. Our findings elucidate a critical mechanism underlying OSCC progression and offer novel therapeutic insights.

## Results

### scRNA-seq and ST profiling of OSCC ecosystems during tumor progression

**Fig. 1:**
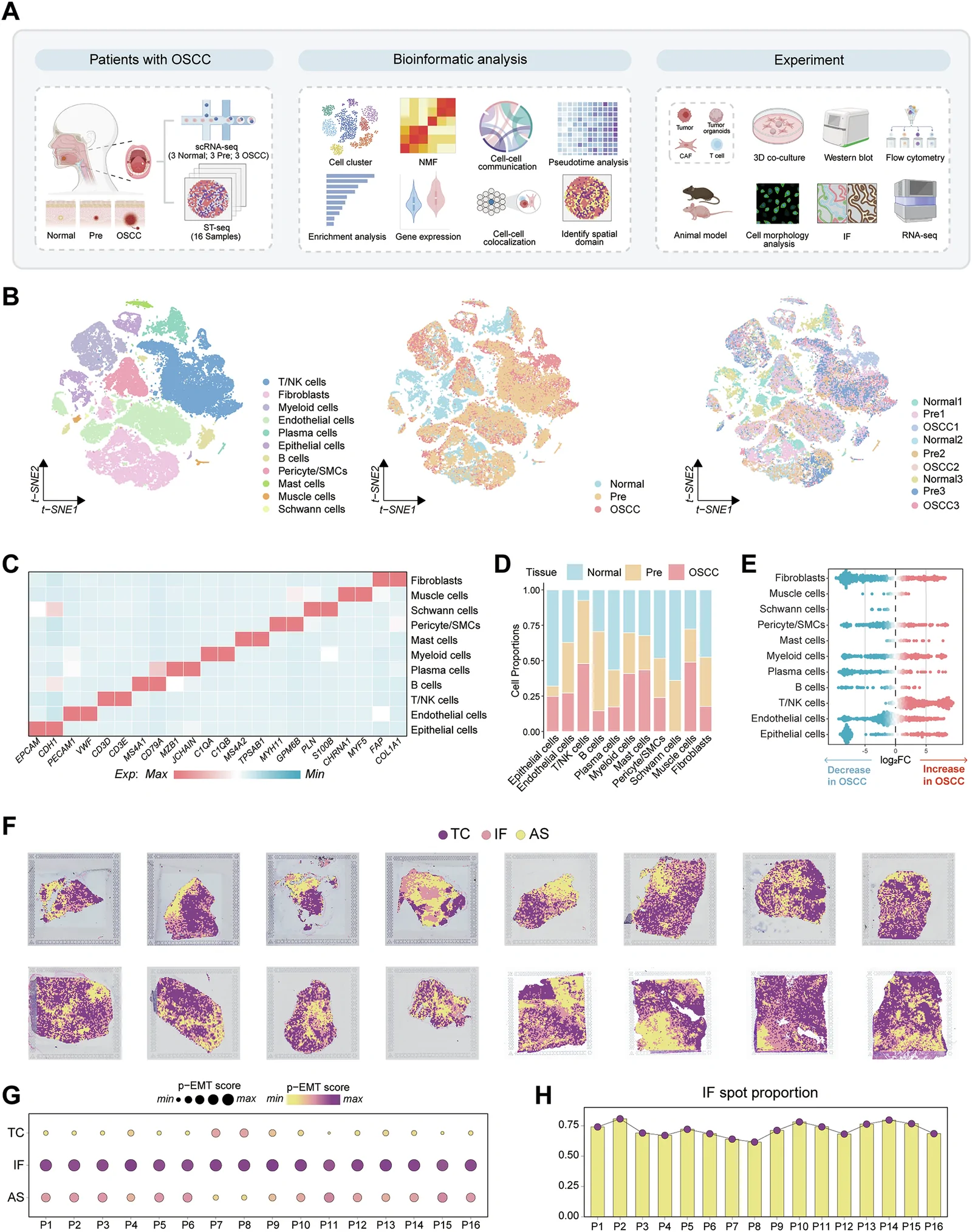
Cellular dynamics in OSCC progression unveiled by scRNA-seq and ST analysis. **A** Schematic diagram of single-cell and spatial transcriptomic analysis as well as experimental design workflow, created with BioRender.com. **B** t-SNE projection of single-cell profiles from the current study, color-coded by major cell types (left), tumor progression stages (middle), and different stages from three patients (right). **C** Average expression profile of canonical marker genes for annotated cell types. **D** Bar plots depicting the proportion of cell types within each tumor progression stage for scRNA-seq profiles. **E** Beeswarm plot showing differences in cell type abundance in log fold change between normal and OSCC, neighborhoods with differential cell abundance at FDR < 0.1 are colored in blue or red, if enriched in normal or OSCC, respectively. **F** Visualization of the spatial distribution of spatial domains in the ST cohort. **G** Dot plots showing the differential expression of p-EMT scores across spatial domains in the ST cohort. **H** Bar plots showing the proportion of points defined as IF domain among the top 25% of points with the highest p-EMT scores in the ST cohort.

To explore the heterogeneity of the tumor ecosystem during the progression of OSCC, we performed scRNA-seq on paired Normal, Pre, and OSCC from three patients (Fig. 1A). All diagnoses were confirmed through meticulous pathological examination. After stringent quality control, we analyzed a total of 56,834 cells across all progression stages, i.e., Normal (*n* = 19,327), Pre (*n* = 19,737), and OSCC (*n* = 17,770) were analyzed. Clustering based on graph-based methods and annotation with canonical cell markers revealed eleven major cell types, which were visualized using t-distributed stochastic neighbor embedding (t-SNE) projections. These included epithelial cells (*n* = 3606), fibroblasts (*n* = 11,277), endothelial cells (*n* = 11,472), T/NK cells (*n* = 15,412), B cells (*n* = 1625), plasma cells (*n* = 2785), mast cells (*n* = 586), muscle cells (*n* = 459), macrophages (*n* = 5232), pericytes/smooth muscle cells (*n* = 4222), and Schwann cells (*n* = 158) (Fig. 1B, C). To assess cell composition dynamics during OSCC progression, we performed differential abundance analysis using MiloR. This analysis revealed significant stage-specific shifts in multiple cell lineages (Fig. 1D, E). Notably, T/NK cells increased from 8% in normal tissues to 48% in OSCC, while other immune populations, including B cells, mast cells, and myeloid cells, also exhibited distinct abundance changes across pathological stages.

To delineate spatial organization and microenvironmental interactions, we assembled a spatial transcriptomics (ST) cohort of 16 samples. We partitioned each tissue into three anatomical compartments: adjacent stroma (AS), invasive front (IF), and tumor core (TC) (Fig. 1F and Supplementary Fig. 1A, see “Methods”). These assignments were validated by pathological review and hematoxylin and eosin (H&E) staining (Supplementary Fig. 1B). The partial epithelial-to-mesenchymal transition (p-EMT) program localized to the invasive front of primary tumors. To substantiate the reliability and quantifiability of our compartmentalization, we calculated p-EMT program across compartments. p-EMT activity was significantly enriched at the IF (Fig. 1G, H).

### Intraepithelial expression heterogeneity in OSCC progression

**Fig. 2:**
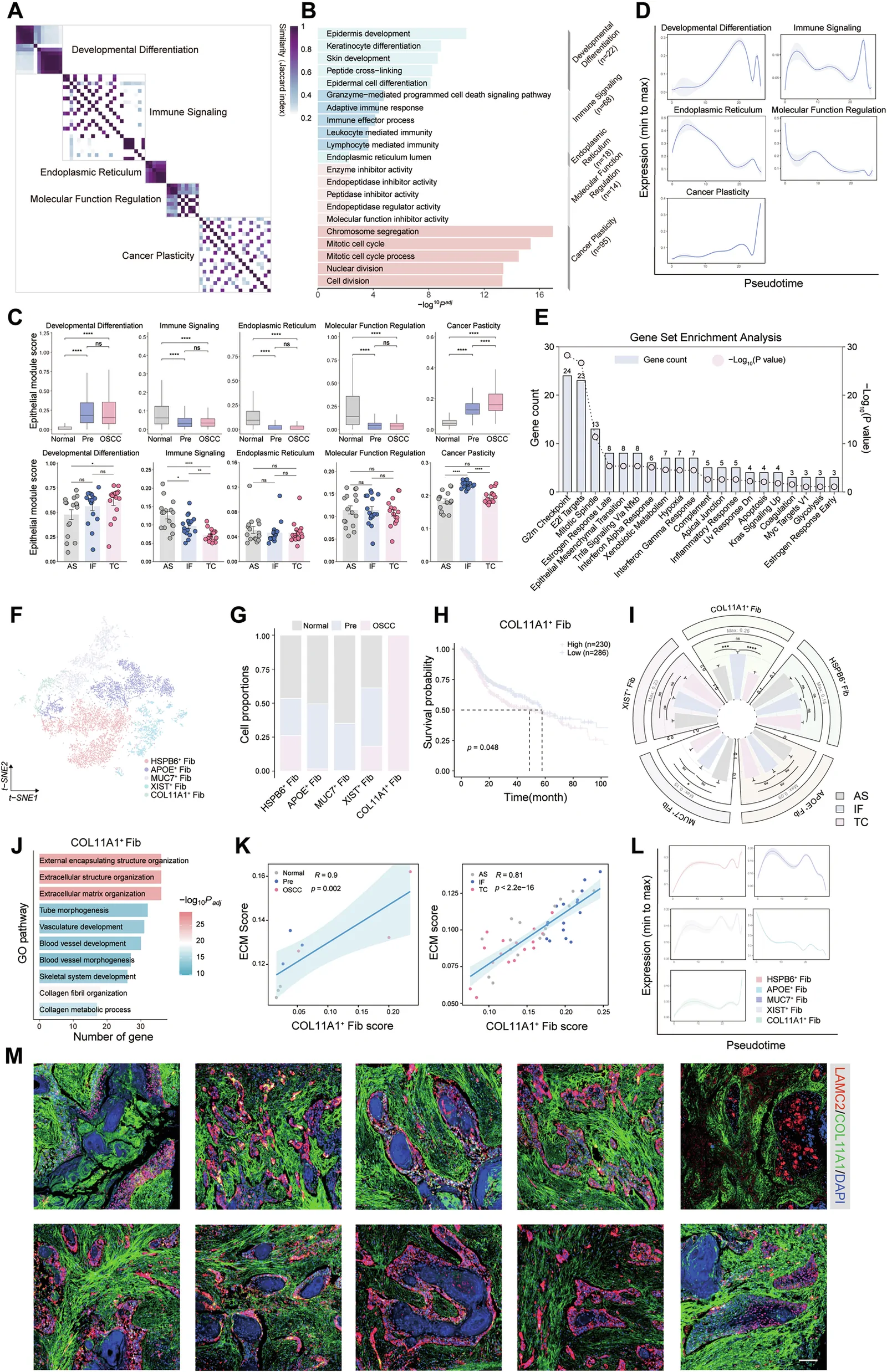
Interpreting the intra-epithelial expression heterogeneity and fibroblast heterogeneity. **A** Hierarchical clustering identified five distinct epithelial modules among epithelial cells from all patients, with similarities between these modules quantified using the Jaccard index. **B** The representative GO pathway terms enriched in each epithelial program. **C** Boxplots show the scores of each epithelial module across tumor progression stages in scRNA-seq (upper row). Bar plots show the spatial variation of the five epithelial program scores across spatial domains in the ST cohort (lower row). **D** Dynamics of epithelial module expression patterns throughout pseudotime in epithelial cell trajectories. **E** Gene set enrichment analysis of cancer plasticity modules. **F** Subclustering of fibroblasts, colored and labeled by subtypes. **G** The proportion of fibroblast subtypes within each tumor progression stage. **H** Kaplan-Meier overall survival curves of TCGA patients stratified by COL11A1^+^ Fib infiltration. **I** Boxplots show the scores of each fibroblast signature across spatial domains in the ST cohort. **J** GO enrichment analysis of differentially expressed genes in COL11A1^+^ Fib. **K** Spearman correlations between COL11A1^+^ Fib signature and ECM score in scRNA-seq (left) and ST cohort (right). Shades represent 95% confidence intervals. **L** Dynamics of fibroblast signatures throughout pseudotime in fibroblast trajectories. **M** Representative images of immunofluorescence characterizing LAMC2-COL11A1 in FFPE sections, with COL11A1 stained green and LAMC2 stained red. Scale bar, 100 µm. Data are presented as the mean ± SEM. ns for not significant; *p < 0.05, **p < 0.01, ***p < 0.001, ****p < 0.0001.

We next characterized expression heterogeneity of epithelial cells during tumor progression. Non-negative matrix factorization (NMF) identified five coherent gene programs co-expressed by epithelial cells (Fig. 2A, Supplementary Fig. 2A, B, and Supplementary Table 1). Gene ontology (GO) analysis annotated these modules as: developmental differentiation (epidermal development and cellular differentiation), immune signaling (adaptive immunity and effector processes), endoplasmic reticulum (ER lumen), molecular function regulation (enzymatic activity modulation), and cancer plasticity (cell division and chromosome segregation) (Fig. 2B). We found that the most significant expression change across the development of OSCC was the dramatic increase of the cancer plasticity module. Spatial analysis localized this module to the IF (Fig. 2C). Differential expression analysis across epithelial subtypes revealed a distinct cluster, C4, characterized by high cancer plasticity activity and CNV scores (Supplementary Fig. 2C).

Using Monocle 2, we constructed a trajectory of epithelial cell progression, which diverged into two branches (Branch 1 and Branch 2) based on differences in transcriptional profiles. The C4 cluster localized to the terminus of Branch 1 and was associated with later pseudotime values (Supplementary Fig. 2D, E). Cancer plasticity module expression roughly increased along pseudotime and peaked at the terminal end of Branch 1 (Fig. 2D and Supplementary Fig. 2F, G). High abundance of the C4 cluster predicted unfavorable overall survival in TCGA bulk RNA-seq data (Supplementary Fig. 2H). Gene set enrichment analysis (GSEA) of the cancer plasticity module revealed enrichment for proliferation, invasion, and EMT pathways (Fig. 2E). In summary, we identified a malignant epithelial C4 cluster characterized by activation of the cancer plasticity module, which may drive the transition from normal epithelial homeostasis to OSCC progression.

### Fibroblast heterogeneity in OSCC progression

We further investigated cell-cell communication across the tumor microenvironment. Epithelial cells and fibroblasts exhibited the strongest interactions (Supplementary Fig. 2I). Fibroblasts underwent distinct phenotypic changes during OSCC progression. Expression-based clustering identified five subpopulations: HSPB6⁺ Fib, APOE⁺ Fib, MUC7⁺ Fib, XIST⁺ Fib, and COL11A1^+^ Fib (Fig. 2F and Supplementary Fig. 2J).

COL11A1^+^ Fib was uniquely enriched in OSCC and associated with unfavorable prognosis (Fig. 2G, H and Supplementary Fig. 2K, L). Similar to the cancer plasticity module, COL11A1^+^ Fib localized to the IF (Fig. 2I). GO analysis revealed enrichment for ECM organization and angiogenesis pathways (Fig. 2J and Supplementary Fig. 2M). There was a positive correlation between the expression of COL11A1^+^ Fib signature and ECM signature across pathological progression and spatial regions (Fig. 2K). These findings suggest that COL11A1^+^ Fib, akin to matrix-associated cancer-associated fibroblast (mCAF) [4]. Trajectory analysis of fibroblast state transition revealed progressive accumulation of COL11A1^+^ Fib signature at the trajectory terminus (Fig. 2L). LAMC2 is a classic marker of IF [17]. Multiplex immunohistochemistry revealed COL11A1⁺ Fib (COL11A1) were localized to the tumor IF (LAMC2) (Fig. 2M).

### PLAU promotes fibroblast activation

**Fig. 3:**
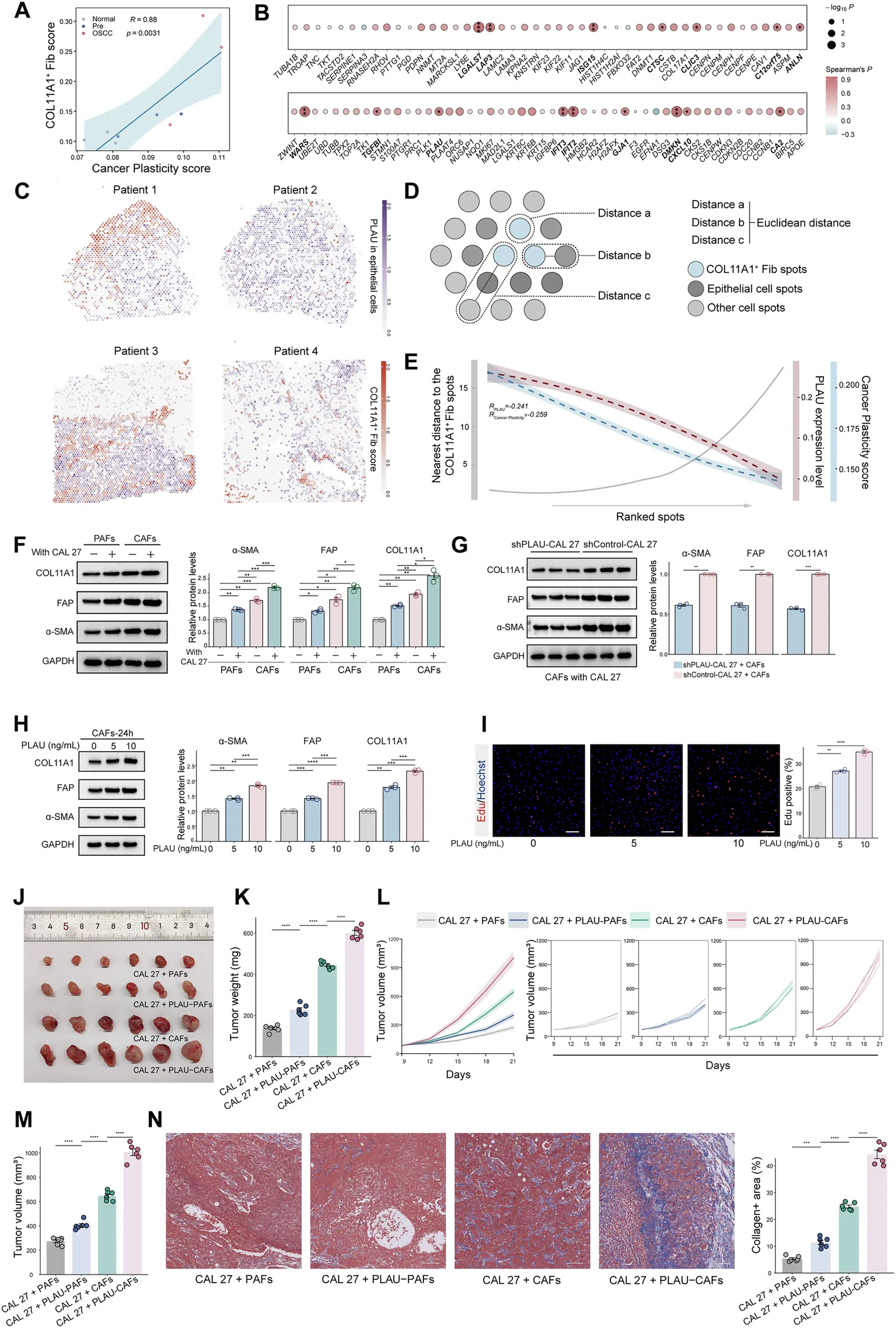
PLAU secreted during OSCC progression promotes fibroblast activation. **A** Spearman correlation between the COL11A1^+^ Fib signature and the cancer plasticity module scores in scRNA-seq. Shades represent 95% confidence intervals. **B** Spearman correlation between the COL11A1^+^ Fib signature and the expression levels of each gene in the cancer plasticity modules in scRNA-seq. **C** ST feature plots illustrating the expression levels and spatial distribution of PLAU in epithelial spots and COL11A1^+^ Fib signature. **D** Schematic diagram illustrating the method of the ST cohort analysis. **E** Spearman correlation between PLAU expression levels and cancer plasticity module scores in epithelial spots, and their nearest distance to COL11A1^+^ Fib spots (RCTD-defined). **F** Western blot analysis and corresponding quantification conducted to detect the protein levels of activated markers, including collagen type XI alpha 1 chain (COL11A1), fibroblast activation protein (FAP), and alpha-smooth muscle actin (α-SMA) in PAFs or CAFs cultured with or without co-culture of CAL 27 cells (*n* = 3). **G** Western blot analysis and corresponding quantification conducted to detect the protein levels of activated markers (COL11A1, FAP, α-SMA) in CAFs after co-culture assay (*n* = 3). **H** Western blot analysis and corresponding quantification conducted to detect the protein levels of activated markers (COL11A1, FAP, α-SMA) in CAFs after treatment with varying concentrations of PLAU protein for 24 h (*n* = 3). **I** Immunofluorescence staining images and quantification of EdU-positive cell proportions after treatment with varying concentrations of PLAU protein for 24 hours (*n* = 3). Scale bar, 100 µm. **J** Photographs of tumors on day 21. **K** Tumor weight analysis on day 21 (*n* = 6). **L** Tumor growth curves (left) and tumor growth curves by group (right). **M** Tumor volume analysis on day 21 for each group (*n* = 6). **N** Masson’s trichrome staining and corresponding quantification of collagen in tumor tissues (*n* = 6). Scale bar, 100 µm. Data are presented as the mean ± SEM. ns for not significant; *p < 0.05, **p < 0.01, ***p < 0.001, ****p < 0.0001.

Since the fibroblast activation coincided with transcriptomic changes in epithelial cells, we examined epithelial-fibroblast interactions during OSCC tumorigenesis. The results showed that among the five epithelial modules, only the cancer plasticity module was positively correlated with COL11A1^+^ Fib signature (Fig. 3A and Supplementary Fig. 3A). The ST data results further validated the cancer plasticity module associated with COL11A1^+^ Fib signature (Supplementary Fig. 3B). We next examined genes within the cancer plasticity module and identified multiple genes significantly correlated with the COL11A1^+^ Fib signature (Fig. 3B and Supplementary Fig. 3C). Through protein-protein interaction (PPI) network analysis, we identified PLAU as the central protein (Supplementary Fig. 3D). Intriguingly, high expression of PLAU was observed in malignant epithelial C4 cluster (Supplementary Fig. 3E).

Further study revealed positive correlation between PLAU expression levels in epithelial cells and the COL11A1^+^ Fib signature (Supplementary Fig. 3F). We also analyzed ST data from four tumor regions. Using scRNA-seq data as a reference, we performed RCTD deconvolution to estimate cell type abundances at each spot. We then calculated Euclidean distances between COL11A1^+^ Fib spots and their nearest epithelial cell spots. A shorter distance between COL11A1⁺ Fib spots and their nearest epithelial cell spots correlated with higher PLAU expression level and cancer plasticity module activity in epithelial cells (Fig. 3C–E). Finally, across multiple cohorts, PLAU was enriched in tumor tissues and its abundance was associated with poor prognosis (Supplementary Fig. 3G–I).

We next experimentally validated fibroblast activation. Co-culture with CAL 27 cells induced higher levels of fibroblast activation markers (α-SMA, FAP, and COL11A1) in paracancerous fibroblasts (PAFs) and CAFs (Fig. 3F). To determine whether PLAU mediates this effect, we established PLAU-knockdown CAL 27 cells (shPLAU#2, Supplementary Fig. 3J). Co-culture with PLAU-knockdown cells reduced the expression of fibroblast activation markers in CAFs compared to co-culture with CAL 27 cells (Fig. 3G). We then treated PAFs and CAFs with recombinant PLAU protein. Dose and time course experiments identified optimal conditions (10 ng/mL, 24 h), which induced elevated fibroblast activation markers (Fig. 3H and Supplementary Fig. 3K–O). EdU staining confirmed increased proliferation of PLAU-treated CAFs (Fig. 3I). To assess the impact on tumor growth in vivo, we co-injected PLAU-treated or untreated CAFs and PAFs, along with CAL 27 cells, into BALB/c nude mice. Tumors co-injected with PLAU-treated CAFs exhibited greater volume and weight compared to untreated groups (Fig. 3J–M). Masson’s trichrome staining showed higher collagen density in tumors from PLAU-treated CAFs (Fig. 3N). These findings indicate that PLAU is essential for CAF activation toward an mCAF phenotype.

### PLAU acts on fibroblast ITGB1

**Fig. 4:**
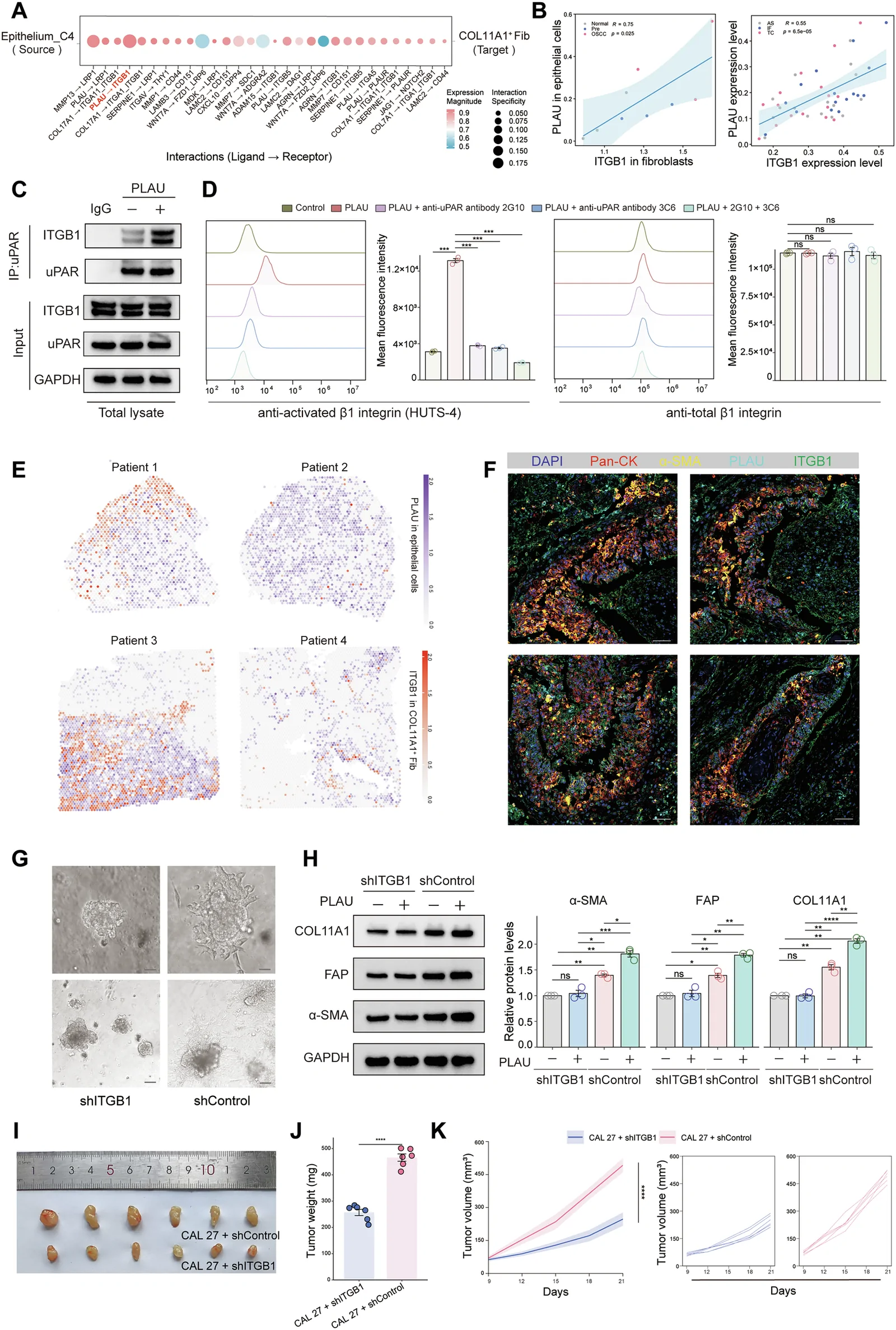
PLAU activates fibroblasts via ITGB1. **A** Dot plot shows the levels of cell-cell communication and the strengths of specific interactions between epithelial cell cluster C4 (source) and COL11A1^+^ Fib (target). **B** Spearman correlation analysis revealing the relationship between PLAU expression levels in epithelial cells and ITGB1 expression levels in fibroblasts in scRNA-seq (left) and ST cohort (right). Shades represent 95% confidence intervals. **C** Co-IP experiment assessing the binding of uPAR to ITGB1 under PLAU protein treatment. **D** Flow cytometry analysis of β1-integrin in CAFs under different treatment conditions (*n* = 3), including active β1-integrin (HUTS-4 antibody, left) and total β1-integrin (anti-CD29 antibody, right). **E** ST feature plots showing the spatial distribution of PLAU expression in epithelial spots (purple) and ITGB1 expression levels in COL11A1^+^ Fib spots. **F** Representative images of mIF for α-SMA, ITGB1, pan-cytokeratin (Pan-CK), PLAU in FFPE sections. Scale bar, 50 µm. **G** Representative image of OSCC organoids co-cultured with CAFs under shControl or shITGB1 treatment conditions. Scale bars, 20 µm (top row), 100 µm (bottom row). **H** Western blot analysis and corresponding quantification assessing the protein levels of activated markers (COL11A1, FAP, α-SMA) in CAFs following treatment with or without PLAU protein for 24 hours, under both shControl and shITGB1 conditions (*n* = 3). **I** Photographs of tumors from different groups on day 21. **J** Tumor weight analysis of different groups on day 21 (*n* = 6). **K** Tumor growth curves (left) and individual tumor growth curves (right). Data are presented as the mean ± SEM. ns for not significant; *p < 0.05, **p < 0.01, ***p < 0.001, ****p < 0.0001.

We next investigated the mechanism by which PLAU promotes fibroblast activation. Through cell-cell interactions between epithelial cell cluster C4 and COL11A1^+^ Fib, we observed the PLAU-ITGB1 pair (Fig. 4A and Supplementary Fig. 4A). To validate this interaction, we integrated multi-omics approaches, which collectively confirmed the correlation between PLAU and ITGB1 expression (Fig. 4B and Supplementary Fig. 4B, C). PLAU signals through urokinase-type plasminogen activator receptor (uPAR), but uPAR lacks intrinsic signaling capacity, necessitating co-receptors such as integrins [14]. We exposed CAFs to PLAU and performed Co-Immunoprecipitation (Co-IP) analysis. PLAU treatment increased the interaction between uPAR and ITGB1 (Fig. 4C). This finding was further validated by flow cytometry using the HUTS-4 monoclonal antibody, which recognizes the active conformation of ITGB1. PLAU-treated cells exhibited a higher proportion of active ITGB1 compared to controls. In contrast, treatment with anti-uPAR antibodies 2G10 (blocking PLAU-uPAR binding) or 3C6 (blocking uPAR-ITGB1 binding) resulted in a lower proportion of active ITGB1 [18]. As a control, these treatments did not alter the expression levels of total ITGB1 (Fig. 4D). Spatial transcriptomics further revealed the spatial proximity of ITGB1 expressing fibroblasts and PLAU expressing epithelial cells (Fig. 4E). Multiplex immunohistochemistry revealed colocalization of PLAU secreted by epithelial cells (marked by Pan-CK) with ITGB1 on the surface of fibroblasts (marked by α-SMA) (Fig. 4F). Notably, PLAU expression in fibroblasts did not correlate with ITGB1 expression (Supplementary Fig. 4D). Together, these findings indicate that the PLAU-ITGB1 interaction occurs between epithelial cells and fibroblasts rather than in an autocrine manner.

We next established ITGB1-knockdown CAFs (shITGB1#3, Supplementary Fig. 4E). Using a three-dimensional organotypic co-culture system of OSCC organoids and fibroblasts, we found that ITGB1 silencing significantly reduced CAF activation and synapse formation (Fig. 4G). Treatment with PLAU protein induced CAF activation, an effect that was abrogated by ITGB1 knockdown (Fig. 4H). To validate the functional importance of ITGB1 in vivo, we co-injected CAL 27 cells with shControl or shITGB1 CAFs subcutaneously into BALB/c nude mice. Mice bearing shITGB1 CAFs knockdown developed smaller tumors compared to those bearing shControl CAFs (Fig. 4I–K). Collectively, these findings demonstrate that ITGB1 is essential for sustaining a tumor-promoting phenotype in vivo. Taken together, these data indicate that PLAU acts on fibroblast ITGB1 to drive CAF activation and tumor progression.

### ITGB1 activates FAK to promote TGFβRI-TGFβRII complex formation

**Fig. 5:**
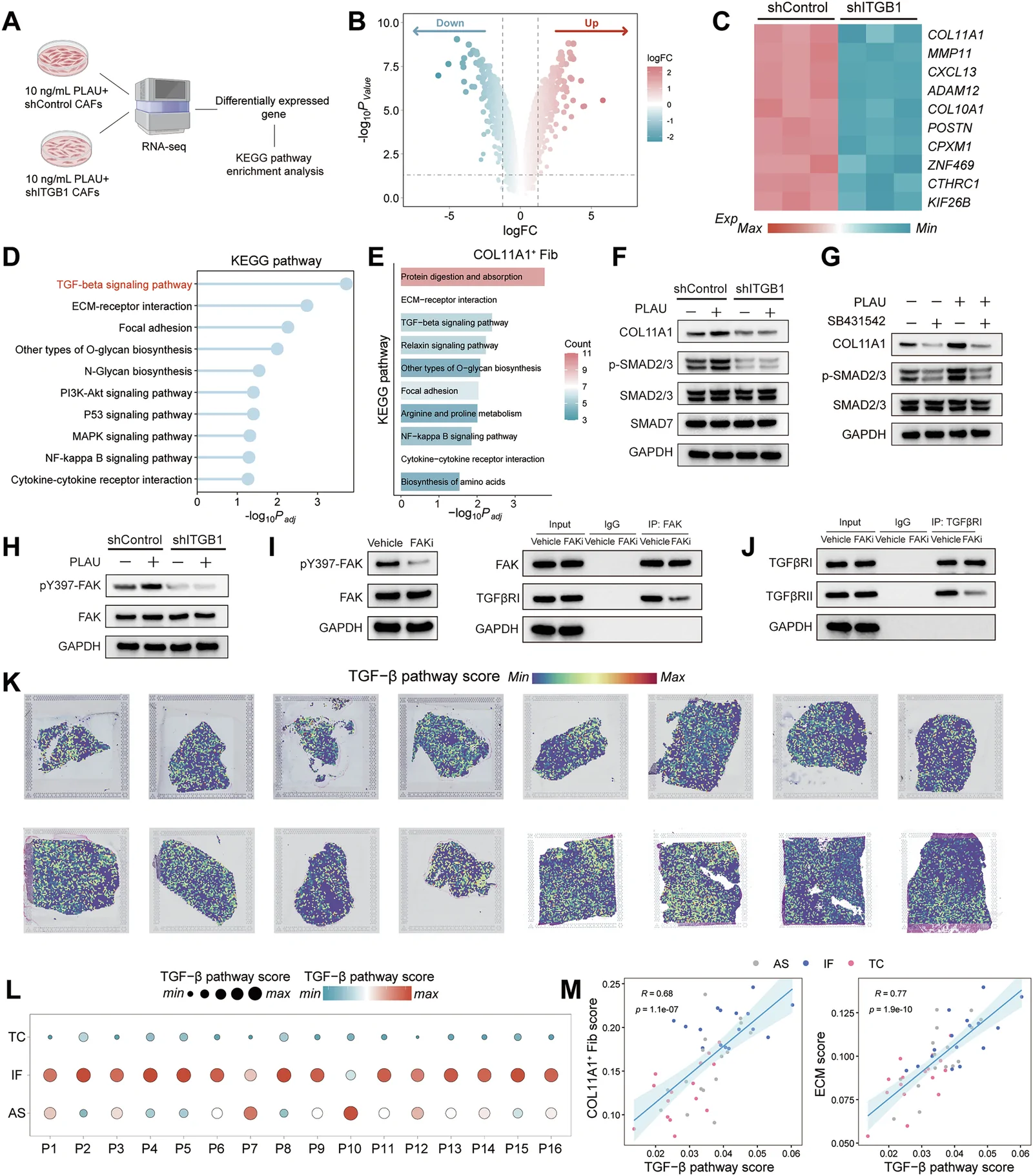
ITGB1 activates FAK to promote TGFβRI-TGFβRII complex formation, thereby activating TGF-β signaling. **A** Experimental design of RNA-seq analysis. **B** Volcano plots showing differentially expressed genes (DEGs) in shControl and shITGB1 CAFs treated with PLAU protein. **C** Heatmap showing the expression levels of COL11A1^+^ Fib top 10 signature genes in RNA-seq. **D** KEGG enrichment analysis in DEGs. **E** KEGG enrichment analysis based on signature genes of COL11A1^+^ Fib from scRNA-seq. **F** Western blotting analysis using the indicated antibodies on cell lysates from different treatment groups. PLAU protein was treated in shITGB1 or shControl CAFs at 10 ng/mL for 24 h. **G** Western blotting analysis using the indicated antibodies on cell lysates from different treatment groups. PLAU (10 ng/mL) and SB431542 (10 µM) were used to treat CAFs for 24 hours. **H** Protein levels of p-FAK and total FAK in shControl or shITGB1 CAFs treatment groups with or without 10 ng/mL PLAU protein treatment for 24 h. **I** Co-IP analysis of the effect of a FAK inhibitor (2 µM) on the interaction between FAK and TGFβRI. **J** Co-IP analysis of the effects of FAK inhibitor (2 µM) on the interactions between TGFβRI and TGFβRII. **K** Spatial feature plots of TGF-β pathway score in ST cohort, spots are colored by the average expression of pathway score. **L** Dot plots showing the differential expression of TGF-β pathway across spatial domains in the ST cohort. **M** Spearman correlations between the COL11A1^+^ Fib signature and the TGF-β pathway score (left), and between the ECM score and the TGF-β pathway score (right) in the ST cohort. Shades represent 95% confidence intervals.

To elucidate the core mechanism by which the PLAU-ITGB1 axis regulates CAF activation, we analyzed the transcriptomes of two groups of CAFs (shControl and shITGB1) treated with PLAU protein. RNA-seq analysis revealed that PLAU treatment upregulated COL11A1^+^ Fib signature genes, while knockdown of ITGB1 blocked their expression (Fig. 5A–C). scRNA-seq and RNA-seq analyses further uncovered activation of the TGF-β signaling pathway (Fig. 5D, E). Subsequent Western blot analysis confirmed that PLAU activates the TGF-β pathway via ITGB1, leading to SMAD2/3 phosphorylation and COL11A1 upregulation, while total SMAD2, SMAD3, and SMAD7 remained unchanged. This activation was rescued by ITGB1 knockdown (Fig. 5F). Additionally, PLAU-induced fibroblast activation was suppressed by the TGF-β pathway inhibitor SB431542 (Fig. 5G). Previous research has established that FAK signaling is initiated by integrin-mediated cell adhesion [19]. ITGB1 promotes FAK phosphorylation at tyrosine 397, thereby increasing FAK kinase activity. Our results showed that PLAU treatment increased FAK phosphorylation, and this activation was rescued by ITGB1 knockdown (Fig. 5H). Furthermore, FAK regulates TGF-β signaling by modulating the interaction between TGF-βRI and TGF-βRII [20]. Using the FAK inhibitor VS-4718 to block FAK kinase activity, Co-IP assays revealed that FAK inhibition not only reduced the interaction between FAK and TGF-βRI (Fig. 5I), but also decreased the association between TGFβRI and TGFβRII (Fig. 5J). Consistently, knockdown of ITGB1 diminished TGFβRI-TGFβRII interactions (Supplementary Fig. 5A). Furthermore, we showed that FAK inhibitor treatment reduced tyrosine phosphorylation on TGFβRI (Supplementary Fig. 5B). Collectively, these data indicate that ITGB1 activates FAK, which in turn promotes TGF-β receptor complex formation, thereby initiating downstream canonical Smad signaling.

Finally, we analyzed the major contributing cells of the TGF-β pathway in the tumor microenvironment. Consistent with experimental findings, COL11A1^+^ Fib exhibited the highest TGF-β pathway activity (Supplementary Fig. 5C, D). ST cohort revealed that TGF-β pathway activity was predominantly enriched at the IF, where it exhibited clear spatial correlation with the COL11A1⁺ Fib signature and the ECM signature (Fig. 5K–M).

### Activation of TGF-β signaling and CREB3L1 drives the transition to COL11A1^+^ Fib

**Fig. 6:**
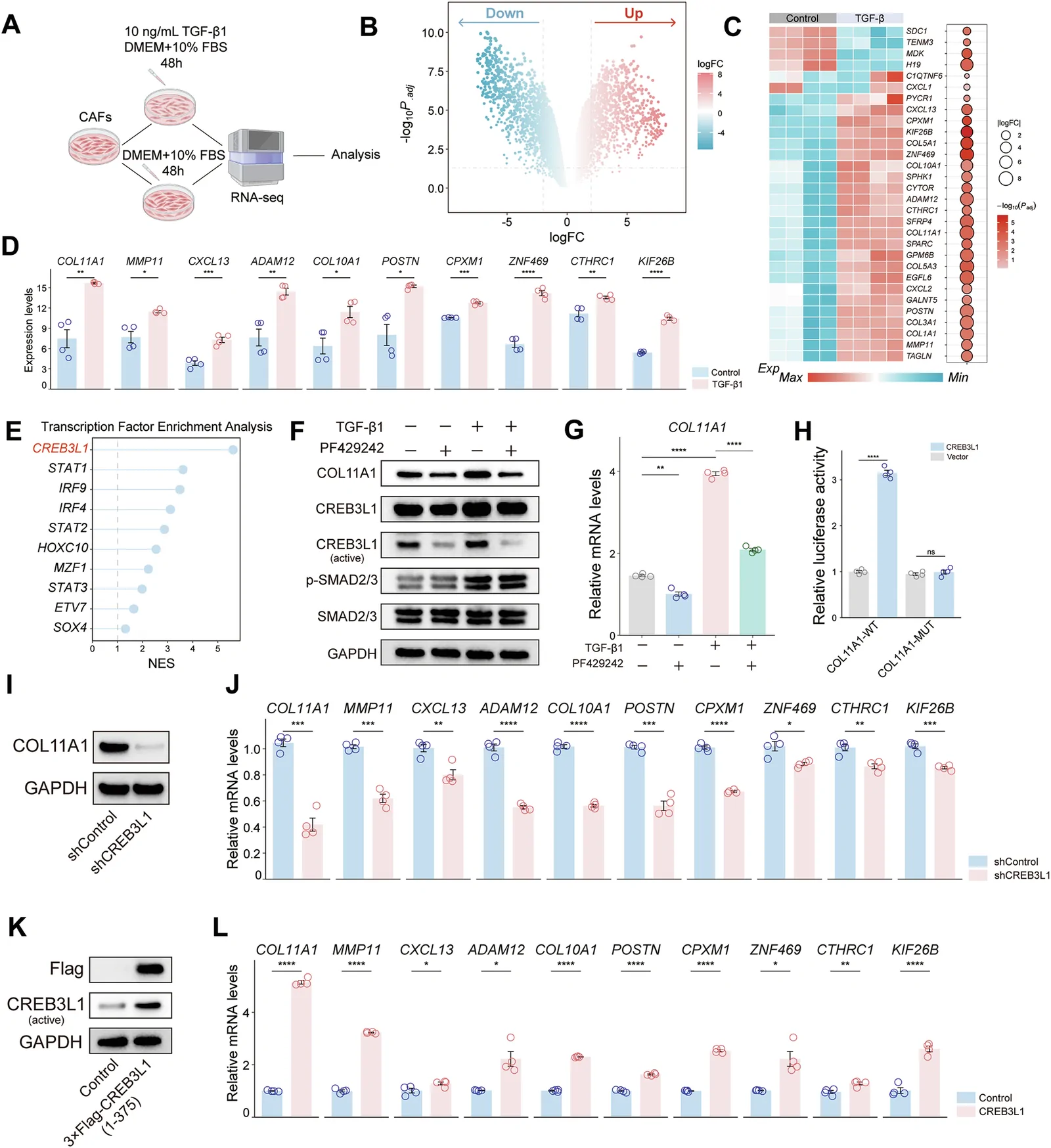
Activation of TGF-β signaling and CREB3L1 drives the transition to COL11A1^+^ Fib. **A** Experimental design of RNA-seq analysis. **B** Volcano plots showing differentially expressed genes (DEGs) in CAFs treated with TGF-β1 compared to controls. **C** Heatmap showing the expression levels of COL11A1^+^ Fib signature genes in RNA-seq analysis. **D** Bar plots show the expression of COL11A1^+^ Fib top 10 signature genes in RNA-seq (*n* = 4). **E** Lollipop plots show normalized enrichment scores (NESs) for the indicated transcription factor-binding motifs. **F** Western blotting images using the indicated antibodies on cell lysates from different treatment groups. TGF-β1 (10 ng/mL) and PF429242 (5 µM) were used to treat in CAFs for 24 h. **G** RT-qPCR profiling of COL11A1 mRNA levels (*n* = 4). **H** Relative luciferase activity of the COL11A1 promoter in CAFs transfected with the indicated constructs (*n* = 4). WT, wild type; Mut, mutant. **I** Western blotting analysis using the indicated antibodies on cell lysates from shControl or shCREB3L1 group. **J** RT-qPCR profiling of COL11A1^+^ Fib top 10 signature genes in shControl or shCREB3L1 group (*n* = 4). **K** Western blotting analysis using the indicated antibodies on cell lysates with or without overexpression of 3×Flag-CREB3L1**. L** RT-qPCR profiling of COL11A1^+^ Fib top 10 signature genes with or without overexpression of CREB3L1 (*n* = 4). Data are presented as the mean ± SEM. ns for not significant; *p < 0.05, **p < 0.01, ***p < 0.001, ****p < 0.0001.

The scRNA-seq and RNA-seq analyses revealed that COL11A1^+^ Fib exhibits enhanced TGF-β pathway activity, suggesting that this pathway is functionally linked to fibroblast activation. To investigate the downstream transcriptional mechanism by which TGF-β signaling induces COL11A1^+^ Fib, we treated CAFs with recombinant TGF-β1 protein (Fig. 6A). Transcriptome sequencing showed that TGF-β1 upregulated the expression levels of COL11A1^+^ Fib signature genes and ECM markers (Fig. 6B–D).

Transcription factor binding motif and regulatory network analysis of COL11A1^+^ Fib identified CREB3L1, STAT1, and IRF9 as candidate regulators (Fig. 6E and Supplementary Fig. 6A, B). We individually knocked down CREB3L1, STAT1, and IRF9 in CAFs and assessed COL11A1 mRNA levels by RT-qPCR (Supplementary Fig. 6C–E). Knockdown of STAT1 or IRF9 did not significantly affect COL11A1 expression (Supplementary Fig. 6F). We therefore examined CREB3L1 activation in fibroblasts. Compared with untreated cells, TGF-β1 treatment elevated levels of cleaved CREB3L1 (active form); CREB3L1 cleavage was inhibited by the S1P/S2P inhibitor PF429242 (Fig. 6F). Concurrently, TGF-β1 promoted COL11A1 expression, and this upregulation was rescued by PF429242 (Fig. 6G). In contrast, phosphorylated SMAD2/3—known to act downstream of TGF-β signaling and regulate COL11A1 expression—increased upon TGF-β1 treatment but was unaffected by PF429242 (Fig. 6F). Furthermore, luciferase reporter assays demonstrated that CREB3L1 activates COL11A1 transcription (Fig. 6H and Supplementary Fig. 6G, H). CREB3L1 knockdown significantly reduced COL11A1^+^ Fib signature gene expression levels (Fig. 6I, J). These data indicate that CREB3L1 plays a critical role in transmitting TGF-β signals to activate COL11A1 transcription. Consistent with this, overexpression of active CREB3L1 induced COL11A1^+^ Fib signature gene expression levels (Fig. 6K, L). Together, these findings demonstrate that TGF-β signaling activates CREB3L1 to specifically induce the COL11A1^+^ Fib activation program.

### Accumulation of COL11A1^+^ Fib at the tumor IF forms an immunosuppressive microenvironment

Given the correlation between COL11A1^+^ Fib and unfavorable clinical outcomes, as well as their established role in ECM remodeling, we sought to determine whether modulating COL11A1^+^ Fib could reshape the immune landscape of OSCC. To this end, we examined the spatial niches of COL11A1^+^ Fib in relation to NK cells, T cells, B cells, and myeloid cells. Spatial analysis revealed colocalization of COL11A1^+^ Fib with T cell clusters at the IF (Supplementary Fig. 7A).

**Fig. 7:**
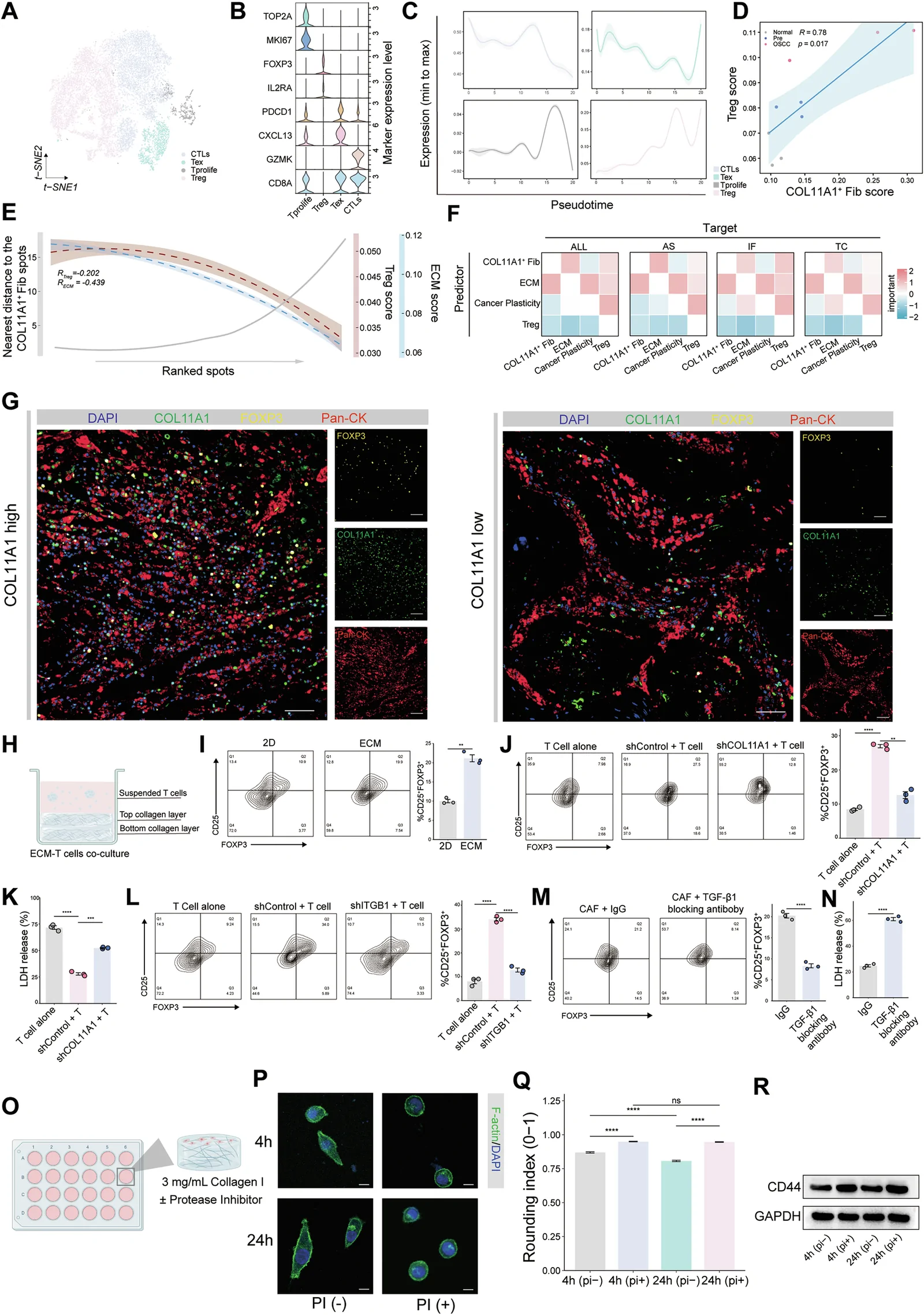
COL11A1^+^ Fib accumulation in tumor IF sculpts an immunosuppressive microenvironment. **A** Subclustering of T cells, colored and labeled by subclusters. **B** Violin plots showing canonical marker-gene expression across T cell subclusters. **C** Pseudotime dynamics of T cell signature scores. **D** Spearman correlation between COL11A1^+^ Fib signature and Treg score in scRNA-seq. Shades represent 95% confidence intervals. **E** Spearman correlation between ECM score and Treg score, and nearest distance to COL11A1^+^ Fib spots. **F** Variable importance for cancer plasticity, Treg, ECM, and COL11A1^+^ Fib signature score. **G** Representative images of mIF for COL11A1, FOXP3, Pan-CK in FFPE sections. Scale bar, 50 µm. **H** Experimental design of collagen-matrix culture system. **I** Flow cytometry analysis and corresponding quantification of Treg cell phenotype (CD25^+^FOXP3^+^) in T cells cultured under 2D or collagen-matrix conditions (*n* = 3). **J** Flow cytometry analysis and corresponding quantification of Treg cell phenotype (CD25^+^FOXP3^+^) in T cells cultured alone or with shControl or shCOL11A1 CAFs (*n* = 3). **K** LDH release assay evaluating T cell-mediated tumor cell cytotoxicity. T cells were pre-cultured alone, with shControl CAFs, or with shCOL11A1 CAFs, then co-cultured with tumor cells (*n* = 3). **L** Flow cytometry analysis of Treg cell phenotype (CD25^+^FOXP3^+^) in T cells cultured alone or with shControl or shITGB1 CAFs, with quantification (*n* = 3). **M** Flow cytometry analysis and corresponding quantification of Treg cell phenotype (CD25^+^FOXP3^+^) in T cells co-cultured with CAFs pre-treated with IgG or TGF-β1 blocking antibody (*n* = 3). **N** LDH release assay in different treatment groups (*n* = 3). **O** Experimental design of collagen-matrix culture system. **P** Immunofluorescence of F-actin (green) in CAL 27 with or without PI treatment; nuclei, blue; F-actin, green. Scale bar, 10 µm. **Q** Cell circularity index; *n* = 406, 406, 384, 425 cells from three independent experiments. **R** Western blot analysis of CD44 in CAL 27 with or without PI treatment at 4 or 24 h. Data are presented as the mean ± SEM. ns for not significant; **p < 0.01, ***p < 0.001, ****p < 0.0001.

We next assessed changes in T cell infiltration during OSCC progression. Notably, Treg infiltration increased at the end of pseudotime, whereas cytotoxic T lymphocyte (CTL) infiltration decreased (Fig. 7A–C). Given the spatial proximity between COL11A1^+^ Fib and T cells, we hypothesized that intratumoral COL11A1^+^ Fib may be associated with T cell infiltration. Consistent with this, correlation analysis revealed a positive association between COL11A1^+^ Fib signature and Treg signature (Fig. 7D, E). We also examined the spatial relationships among these signature markers. COL11A1^+^ Fib, ECM components, cancer plasticity, and Treg were spatially interconnected at the IF (Fig. 7F and Supplementary Fig. 7B). Multiplex IHC further confirmed that high COL11A1 expression regions exhibited increased Treg (FOXP3) infiltration (Fig. 7G). These results suggest that COL11A1⁺ Fib are associated with local Treg accumulation. We further confirmed this finding in vitro. We isolated CD3⁺ T cells from healthy donors, stimulated them with CD3/CD28, and embedded them in a collagen matrix or plated them on 2D dishes (Fig. 7H). After four days, the proportion of CD25⁺ FOXP3⁺ T cells was significantly higher in the 3D matrix than in 2D culture, suggesting that the ECM can increase CD4⁺ T cell differentiation into Treg (Fig. 7I and Supplementary Fig. 7C). Given that COL11A1^+^ Fib are major producers of ECM components, we next investigated whether they also promote T cell differentiation in the context of antitumor immunity (Supplementary Fig. 7D). T cells co-cultured with shControl CAFs showed increased CD4⁺ T cell differentiation into Treg and reduced cytotoxicity against tumor cells, whereas knockdown of COL11A1 in CAFs restored their killing capacity (Fig. 7J, K). Given that ITGB1 is the upstream receptor mediating PLAU-induced fibroblast activation, we next investigated the role of ITGB1 in fibroblast-mediated antitumor immunity. When CAFs (shControl or shITGB1) were co-cultured with T cells, fibroblasts promoted CD4⁺ T cell differentiation into Treg, an effect abrogated by ITGB1 knockdown, demonstrating that ITGB1 is crucial for fibroblast-mediated immunosuppression (Fig. 7L). Finally, we wanted to explore whether TGF-β signaling also contributes to CAF-mediated Treg induction. We inhibited TGF-β signaling in the co-culture system using a TGF-β blocking antibody. This treatment attenuated CD4⁺ T cell differentiation into Treg and enhanced T cell cytotoxicity (Fig. 7M, N). These results suggest that TGF-β signaling is involved in CAF-T cell crosstalk and Treg induction. However, whether the TGF-β ligands originate from CAFs or T cells in this context requires further investigation.

We hypothesized that inhibiting ECM degradation in vitro would increase local matrix density. Adding protease inhibitors (PI) to the collagen matrix induced actin cytoskeleton deformation in CAL 27 cells and increased CD44 expression, suggesting enhanced invasion and stemness (Fig. 7O–R).

### ITGB1 and COL11A1^+^ Fib as potential biomarkers for immunotherapy

**Fig. 8:**
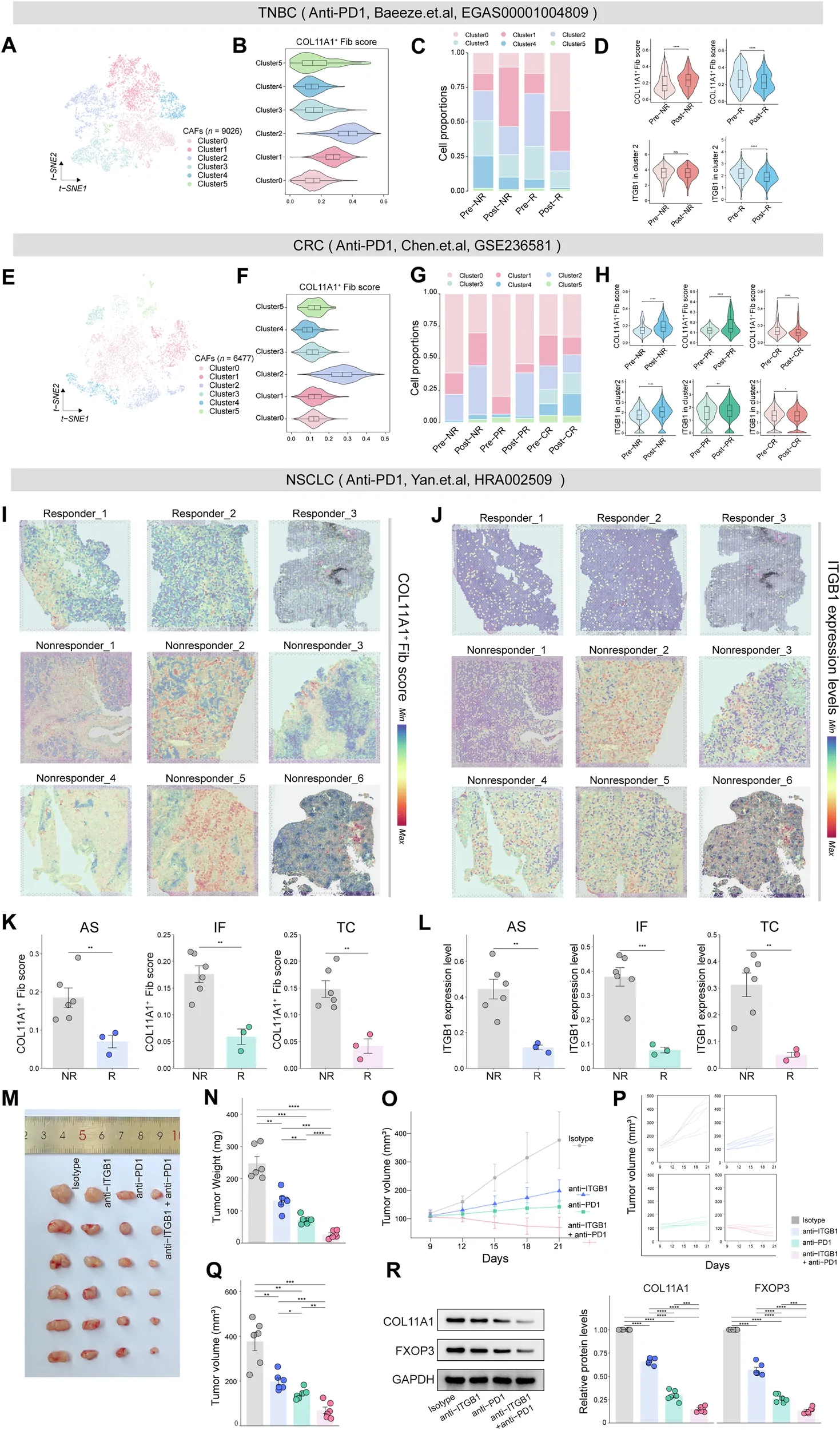
ITGB1 and COL11A1^+^ Fib as potential biomarkers for immunotherapy. **A** Subclustering of fibroblasts in TNBC cohort, colored and labeled by subtypes. **B** Violin plot showing the COL11A1^+^ Fib signature across various fibroblast subtypes in TNBC cohort. **C** Bar plots depicting the proportion of fibroblast subclusters within pre-treatment and post-treatment tissues from patients with different responses to immunotherapy in the TNBC cohort. **D** Violin plot showing the COL11A1^+^ Fib signature and ITGB1 expression levels within pre-treatment and post-treatment tissues from patients with different responses to immunotherapy in the TNBC cohort. **E** Subclustering of fibroblasts in CRC cohort, colored and labeled by subtypes. **F** Violin plot showing the COL11A1^+^ Fib signature across various fibroblast subtypes in CRC cohort. **G** Bar plots depicting the proportion of fibroblast subclusters within pre-treatment and post-treatment tissues from patients with different responses to immunotherapy in the CRC cohort. **H** Violin plot showing the COL11A1^+^ Fib signature and ITGB1 expression levels within pre-treatment and post-treatment tissues from patients with different responses to immunotherapy in the CRC cohort. **I** Spatial feature plot of COL11A1^+^ Fib signature in the NSCLC cohort. **J** Spatial feature plot of ITGB1 expression levels in the NSCLC cohort. **K** Bar plots showing the COL11A1^+^ Fib signature scores across different spatial domains in the NSCLC cohort. **L** Bar plots showing the ITGB1 expression levels across different spatial domains in the NSCLC cohort. **M** Photographs of tumors on day 21. **N** Tumor weight analysis of different treatment groups on day 21 (*n* = 6). **O** Tumor growth curves of different treatment groups (*n* = 6). **P** Individual tumor growth curves for each treatment group (*n* = 6). **Q** Tumor volume analysis of different treatment groups on day 21(*n* = 6). **R** Western blot analysis and corresponding quantification of COL11A1 and FOXP3 protein levels in tumors after receiving different treatment (*n* = 6). Data are presented as the mean ± SEM. ns for not significant; *p < 0.05, **p < 0.01, ***p < 0.001, ****p < 0.0001.

The tumor immune microenvironment is a pivotal determinant of immune checkpoint blockade (ICB) therapy responses. The close association of COL11A1^+^ Fib with the immunosuppressive microenvironment prompted us to investigate their role in ICB response. To explore the predictive value of COL11A1^+^ Fib for immunotherapy, we analyzed cohorts across different cancer types. First, we obtained scRNA-seq data from triple-negative breast cancer (TNBC) patients treated with an anti-PD-1 antibody. t-SNE projection revealed six fibroblast subsets, with subset 2 exhibiting COL11A1^+^ Fib signature. In responders, both the abundance and COL11A1^+^ Fib signature decreased after immunotherapy, whereas non-responders showed no change. ITGB1 expression levels were also significantly reduced in fibroblasts from responders (Fig. 8A–D and Supplementary Fig. 8A). Next, we analyzed scRNA-seq data from colorectal cancer (CRC) patients treated with an anti-PD-1 antibody. t-SNE projection identified six fibroblast subsets, with subset 2 highlighting COL11A1^+^ Fib characteristics. Notably, non-responders and partial responders exhibited increased abundance and COL11A1^+^ Fib signature after immunotherapy, whereas responders showed a decrease. ITGB1 expression levels were significantly reduced in responders but increased in non-responders and partial responders (Fig. 8E–H and Supplementary Fig. 8B). Finally, we analyzed spatial transcriptomic data from non-small cell lung cancer (NSCLC) patients treated with an anti-PD-1 antibody. In patients with high response rates to immunotherapy, the COL11A1^+^ Fib signature and ITGB1 expression levels were significantly downregulated in TC, IF, and AS. Patients with low ITGB1 expression levels and low COL11A1^+^ Fib signature showed significantly higher response rates to immunotherapy (Fig. 8I–L and Supplementary Fig. 8C).

In summary, these findings support the value of COL11A1^+^ Fib and ITGB1 as potential biomarkers for immunotherapy response. Given these associations, we next investigated whether targeting ITGB1 could enhance immunotherapy response in an OSCC model. We found that anti-ITGB1 treatment in combination with anti-PD-1 significantly reduced COL11A1^+^ Fib abundance and alleviated immunosuppression, thereby enhancing the response rate to OSCC immunotherapy (Fig. 8M–R).

## Discussion

Fibroblasts play a critical role in OSCC development and progression. However, the molecular mechanisms that drive fibroblast activation and functional remodeling within the tumor microenvironment have remained largely unknown. In this study, we employed an integrated multi-omics approach to elucidate the critical cellular evolutionary trajectories, cellular heterogeneity, and intercellular interactions during OSCC development. We focused on the crosstalk between epithelial cells and fibroblasts, and revealed that during malignant transformation, malignant epithelial cells secrete PLAU, which activates the TGF-β pathway via ITGB1 on fibroblasts, driving their conversion into COL11A1^+^ Fib.

NMF uncovered five gene modules that act as distinct molecular players during tumor development. These modules reveal intrinsic regulators of dynamic cell-state transitions. In particular, the cancer plasticity module, characterized by genes driving cell cycle progression and cellular invasion and spatially localized to the IF evokes a cascade of alterations in key cancer-related pathways, such as cell cycle control and epithelial-to-mesenchymal transition thereby fueling OSCC progression [21–23]. The tumor microenvironment encompasses a heterogeneous population of fibroblasts with either pro-tumorigenic or anti-tumorigenic roles [24–26]. We discovered a key COL11A1^+^ Fib population in the progression of OSCC, characterized by the enrichment of collagen secretion pathways.

The interaction between epithelial cells and fibroblasts is central to the formation of the Epi-Fib niche. In the cancer plasticity module, we identified a core gene, PLAU, whose expression progressively increases during OSCC progression. Previous studies have reported that PLAU promotes tumor progression in various cancer types and also facilitates the conversion of fibroblasts into inflammatory cancer-associated fibroblast (iCAF) [27–29]. Although PLAU is a well-known agonist of uPAR, uPAR lacks intrinsic signaling capacity, and downstream signaling following PLAU-uPAR binding requires additional co-receptors [18]. In the present study, we demonstrate that PLAU acts through uPAR on fibroblasts to promote uPAR-ITGB1 interaction, leading to ITGB1 activation. Although the role of TGF-β signaling in cancer progression is well established, the crosstalk between integrins and the TGF-β pathway remains poorly documented. Previous studies have shown that TGF-β activates focal adhesion kinase (FAK) through integrin β1 or β3, thereby activating p38 MAPK [30]. In the present study, we found that integrin β1-FAK is involved in the canonical Smad-dependent pathway. Furthermore, we demonstrated that PLAU induces FAK phosphorylation via ITGB1, and FAK kinase activity is required for the tyrosine phosphorylation of TGFβRI, which enhances TGFβRI-TGFβRII binding affinity, increases responsiveness to TGFβ exposure, thereby activating downstream Smad-dependent pathways. In COL11A1^+^ Fib, we identified CREB3L1 as a downstream mediator of TGF-β signaling, which was demonstrated by the significant alleviation of TGF-β-mediated COL11A1 expression following PF429242 treatment, as well as by dual-luciferase reporter assays. Overexpression and knockdown of CREB3L1 both alter the expression of COL11A1^+^ Fib markers.

At the tumor IF, we identified a niche involving the cancer plasticity module, COL11A1^+^ Fib, ECM, and Treg. ECM remodeling, along with cytokine signals and fibroblast-T cell interactions, collectively drive T cell differentiation into immunosuppressive Tregs, thereby impeding CD8⁺ T cell infiltration. This process establishes a dual immunosuppressive barrier of COL11A1^+^ Fib-ECM and Tregs at the tumor IF, creating an immune-excluded microenvironment that restricts immune cell infiltration and activation, representing the loss of the host’s “last line of defense” against the tumor [31, 32]. These findings highlight the complexity of the Epi-COL11A1^+^ Fib-Treg interactions and further enhance our understanding of the dynamic interplay of COL11A1^+^ Fib.

These findings highlight the mechanisms of immune suppression centered on COL11A1^+^ Fib. Thus, blocking the activation of fibroblasts by epithelial cells, which may be the starting point of malignant progression, offers a potential therapeutic strategy for OSCC. However, targeting epithelial cells directly with PLAU inhibitors is challenging due to the presence of the extracellular matrix barrier and the risk of tumor cell escape. Thus, we identified ITGB1 as a key component of the crosstalk between fibroblasts and the tumor microenvironment. Targeting COL11A1^+^ Fib by inhibiting ITGB1 may offer a two-for-one benefit [33, 34]. Our study also explored the close association between COL11A1^+^ Fib and ICB treatment response. We observed that after ICB treatment, responders exhibited lower COL11A1⁺ Fib infiltration compared to non-responders, as well as compared to pre-treatment levels. Moreover, ITGB1 expression was significantly reduced in responders after ICB treatment. These results underscore the potential of COL11A1^+^ Fib and ITGB1 as predictive biomarkers for ICB treatment response [35, 36].

Our study may be limited by the finite sample size. Enrolling a larger cohort of samples may reveal novel mechanisms underlying the malignant progression of OSCC. In summary, we elucidate that PLAU-ITGB1-TGF-β signaling-driven fibroblast activation is a pivotal process in the formation of the Epi-COL11A1^+^ Fib-Treg dual immunosuppressive barrier niche. Our results may have profound significance for understanding cancer development and for the prevention and clinical treatment of OSCC.

## Material and methods

### Cell culture on collagen I layers

To simulate the extracellular matrix environment, collagen type I solution (40125ES50, Yeasen) was diluted to 3 mg/mL. For cell morphology assessment, 500 µl of collagen solution was added to each well of a 24-well plate and polymerized for 60 min at 37 °C. Cells were then seeded at a density of 1 × 10⁵ cells/100 µl onto the collagen gel in medium containing 10% FBS and cultured for 24 h to allow adhesion. Subsequent treatments were applied in medium containing 1% FBS. On the day of confocal imaging, the gel was carefully flipped for staining. F-actin was stained with iFluor™ 488 phalloidin (40736ES75, Yeasen) and nuclei with DAPI (40728ES03, Yeasen) [37]. For the Collagen treatment of T cells experiment, 250 µl of collagen solution was added to a well of a 24-well plate and polymerized at 37 °C for 60 min. A collagen solution containing 1 × 10⁶ CD3⁺ T cells was layered on top and polymerized at 37°C for an additional 60 min. Finally, 750 µl of culture medium was added [33]. After 72 h of culture, cells were extracted from the gel using 3 mg/mL collagenase at 37 °C in a shaking incubator for 60 min. Following washing and filtration, cells were resuspended in HBSS with 2% FBS for subsequent surface protein staining.

### Cell proliferation assay

Cell proliferation was assessed using an EdU kit (FNCK111, FineTest). Cells were seeded in a 96-well plate at 0.8 × 10⁵ cells/well and cultured for 24 h. The experimental group was treated with PLAU protein. After 24 h of treatment, the culture medium was replaced with EdU medium and incubated for 6 h. Following PBS rinses, cells were treated with Click Additive Solution for 30 min. Nuclei were stained with Hoechst 33342 to determine the proliferation rate, and images were captured using a fluorescence microscope. All procedures followed the manufacturer’s instructions.

### Isolation and culture of fibroblasts

Oral cancer tissues were freshly isolated from post-surgical OSCC specimens and preserved in DMEM at 4 °C. After PBS washing, tissues were minced into 1 mm³ fragments and digested with 1 mg/mL collagenase (HY-E70005A, MedChemExpress) at 37 °C in a shaking incubator for 60 min. Post centrifugation and filtration, cells were resuspended, seeded into cell culture bottle, and cultured for 30 min. Nonadherent cells were removed via medium exchange. Homogeneous CAFs were obtained after 3 passages. PAFs were isolated from paracancerous tissues using identical protocols.

### Isolation and culture of T cells

Human peripheral blood mononuclear cells (PBMCs) were isolated from healthy donors via density-gradient centrifugation using Peripheral blood lymphocyte separation tube (601851, NEST Biotechnology) and purified with anti-CD3 MicroBeads (130-097-043, Miltenyi Biotec). Cells were cultured in RPMI-1640 medium (KGL1505-500, Keygen BioTECH) supplemented with 10% FBS (FBS-300, Jin Yuan Kang Biotechnology), 10 µg/mL anti-human CD3/CD28 mAb (UA090033, UA BIOSCIENCE), 10 ng/mL IL-2 (UA090033, UA BIOSCIENCE), and 0.05 mM β-mercaptoethanol (HY-Y0326, MedChemExpress).

### T cells co-culture model of CAFs and flow cytometry analysis

shControl, shITGB1, or shCOL11A1 CAFs were seeded in 24-well plates at 1 × 10⁵ cells/well and cultured to 75% confluence. To validate the role of TGF-β pathway, we used a TGF-β1 blocking antibody (MA5-23795, Thermo Fisher Scientific) to neutralize TGF-β1 activity, with IgG antibody as a control. Subsequently, 1 × 10⁶ CD3⁺ T cells were added per well and co-cultured in IL-2/β-mercaptoethanol-supplemented medium. After 72 h, T cells were harvested, filtered, and stained for surface markers. Cells were collected in HBSS (KGL2202-500, Keygen BioTECH) with 2% FBS and incubated with the following antibodies: CD3 FITC (344803, BioLegend), CD4 PE/Cyanine7 (357409, BioLegend), CD8 PerCP/Cyanine5.5 (344709, BioLegend), CD25 APC (356109, BioLegend), and FOXP3 BV421™ (320123, BioLegend). Experiments were repeated three times using T cells from independent healthy donors.

To assess β1-integrin activation, 1 × 10⁵ CAFs were treated as follows: (1) untreated control; (2) recombinant PLAU (10 ng/mL); (3) anti-uPAR antibody clone 2G10 (ZRB1224, Sigma-Aldrich); (4) anti-uPAR antibody clone 3C6 (MABC89, Sigma-Aldrich); and (5) combination of clone 2G10 and 3C6. Cells were then stained with HUTS-4 antibody (FCMAB389F, Sigma-Aldrich), which specifically recognizes the activated conformation of β1-integrin. As a control for total β1-integrin expression, CAFs were also stained with anti-CD29 antibody (130-123-935, Miltenyi Biotec).

### LDH assay

To evaluate T cell-mediated tumor cell cytotoxicity, T cells were first pre-cultured alone or with CAFs under indicated conditions for 72 h. T cells were then harvested, washed, and co-cultured with tumor cells at a T cell-to-tumor cell ratio of 10:1 for 24 hours. LDH release was measured using the LDH Cytotoxicity Assay Kit (HY-K1090, MedChemExpress) according to the manufacturer’s instructions.

### Lentiviral transduction and plasmid transfection

Short-hairpin RNA (shRNA) sequences targeting human ITGB1, IRF9, CREB3L1, STAT1, COL11A1, and PLAU (Supplementary Table 3) were designed, and the lentiviruses were packaged by GeneChem Co., Ltd. (Shanghai, China). Lentiviral transduction was performed according to the manufacturer’s protocol, and stable cell lines were selected with 1 µg/mL. puromycin (AB11L241, Life-iLab, China). Knockdown efficiency was confirmed by Western blot. The pCMV-3×FLAG-CREB3L1 (1-375) and pCMV-3×FLAG-TGFβRI plasmids were constructed by Sangon Biotech Co., Ltd. (Shanghai, China).

### Transwell co-culture assay

Tumor cells (1 × 10⁵ cells/well) were seeded in the upper chamber of 24-well Transwell inserts (725131, NEST Biotechnology). CAFs (5 × 10⁴ cells/well) were plated in the lower chamber in DMEM supplemented with 10% FBS. After co-culture, CAFs in the lower chamber were harvested for subsequent analysis.

### Animal models

To explore Epi-Fib crosstalk, CAFs or PAFs (cultured with 10 ng/mL PLAU for 24 h or control, 5 × 10⁵) were combined with 5 × 10⁵ CAL 27 cells and injected into BALB/c nude mice. On day 21, mice were euthanized, and tumors were harvested for analysis. To elucidate the role of ITGB1 in CAFs, CAFs (shControl or shITGB1, 5 × 10⁵) were mixed with 5 × 10⁵ CAL 27 cells and injected into BALB/c nude mice. Mice were euthanized on day 21, and tumors were collected for analysis. For combined immunotherapy studies, 1 × 10⁶ SCC 7 cells were implanted subcutaneously into C3H mice and assigned to different groups. When tumors reached ~100 mm³ (around 8 days post-implantation), treatments began: intraperitoneal injections of anti-PD-1 (BE0273, BioXcell), anti-Integrin β1, a combination of both, or isotype control antibody at 100 μg per mouse, administered every 3 days for four doses until experiment completion. Four-week-old BALB/c nude mice and C3H mice were sourced from Vital River Laboratory Animal Technology Co., Ltd. Mice were housed in individually ventilated cages within a specific pathogen-free barrier at the Shandong Provincial Hospital Affiliated to Shandong First Medical University. All experiments adhered to the National Institutes of Health guidelines for laboratory animal care and were approved by the Animal Care and Use Committee of Shandong Provincial Hospital Affiliated to Shandong First Medical University. This study was approved by Shandong Provincial Hospital Affiliated to Shandong First Medical University (NO.2024-242).

### Multiplex immunofluorescence (mIF) and Masson trichrome staining

Tumor tissues from humans and mice were embedded in paraffin, sectioned, deparaffinized, rehydrated, and subjected to antigen retrieval. After PBS washing, sections were permeabilized with enhanced immunostaining permeabilization solution (P0097, Beyotime) and blocked with 10% goat serum. Sections were incubated overnight at 4 °C with primary antibodies against LAMC2 and COL11A1 (two-marker panel), Pan-CK, FOXP3, and COL11A1 (three-marker panel), or Pan-CK, PLAU, α-SMA, and ITGB1 (four-marker panel), primary antibodies were used at 1:200 dilution followed by secondary antibody incubation and fluorescein-conjugated tyramide signal amplification (TSA). Microwave treatment was applied to remove antibodies between staining cycles, and blocking was repeated. Nuclei were stained with DAPI, and images were scanned using a digital slide scanner. Masson trichrome staining was performed on paraffin-embedded sections using a Masson trichrome staining kit (C0189S, Beyotime) according to the manufacturer’s instructions.

### Bulk RNA sequencing

After PBS washing, cells were collected and total RNA was extracted. RNA concentration and purity were assessed using a NanoDrop spectrophotometer. Samples passing quality control were used for sequencing library construction with the TruSeq RNA Library Prep Kit (Illumina) and subsequent sequencing.

### Cell culture

Human oral squamous cell carcinoma (OSCC) cell line CAL 27 was purchased from Procell Co., Ltd, and mouse OSCC cell line SCC 7 was obtained from Xiamen Immocell Biotechnology Co., Ltd. Both cell lines were cultured in DMEM (PM150212, Procell) supplemented with 10% FBS (FBS-300, Jin Yuan Kang Biotechnology). They were maintained at 37 °C in a 5% CO₂ incubator and authenticated via Short Tandem Repeat (STR) profiling. No commonly misidentified cell lines were used.

### Organoid and fibroblast co-culture model

Patient-derived organoids from OSCC patients were established as described [3]. Organoids were collected in pre-chilled PBS and mechanically dissociated by repeated pipetting. Subsequently, they were digested with trypsin at 37 °C for 3 min. To assess the role of ITGB1 in CAFs, CAFs (shControl or shITGB1, 1 × 10⁴) were mixed with 1 × 10⁴ tumor organoid cells and combined with Matrigel (KE100-10; K2 Oncology). The mixture was then seeded into a pre-warmed 48-well plate (3548, Corning). Once the Matrigel solidified, 500 µl of DMEM was added, and the cultures were maintained for 48 h.

### Human tissue sample collection

All patient samples were collected with approval from the Ethics Committee of Shandong Provincial Hospital Affiliated to Shandong First Medical University (NO.2024-115). Informed consent was obtained from each patient, and clinical information was gathered from medical records. Fresh tissues, including OSCC and adjacent normal tissues, were collected immediately post-surgery from patients who had not received prior therapy.

### Confocal images were analyzed using ImageJ

For cell morphology assessment, individual cells in F-actin-stained images were segmented using Cellpose, and single-cell shape circularity was quantified using ImageJ.

### Western blot

Cells were lysed in RIPA buffer containing protease and phosphatase inhibitor cocktails (R0010, Solarbio). Protein concentration was measured using a BCA Protein Assay Kit (PC0020, Solarbio). Cell lysates were separated via precast 4-20% gradient SDS-polyacrylamide gel electrophoresis (abs9604, absin) and transferred to PVDF membranes (IPVH00010, Millipore). After blocking with 5% skim milk (D8340, Solarbio), primary antibodies were diluted in antibody diluent (K1803, HUABIO) and incubated with the membranes overnight at 4 °C, followed by incubation with HRP-conjugated secondary antibody for 1 h at room temperature. Protein bands were visualized using enhanced chemiluminescence (ECL) detection (ED0015-B, Shandong Sparkjade Biotechnology Co., Ltd.) in a ChemiDoc MP imaging system and quantified using ImageJ. The original data for all blots are provided in the supplementary materials.

### Real-time quantitative reverse transcriptase PCR (Real-time RT-PCR) and primer sequences

Total RNA was extracted using an RNA Extraction Kit (AG21023) and dissolved in RNase-free water. cDNA was synthesized using a Reverse Transcription Premix Kit (AG11705), followed by RT-qPCR using SYBR Green Pro Taq HS Premix (AG11701) on a LightCycler 480 system (Roche). All reagents were purchased from Accurate Biotechnology (Hunan), Changsha, China. The relative expression levels of target genes, normalized to GAPDH, were calculated using the 2^−ΔΔCt method. Primer sequences are listed in Supplementary Table 4.

### Coimmunoprecipitation assays (Co-IP)

For the Co-IP assay, cells were lysed and the lysates were incubated with antibody, or IgG isotype control at 4 °C overnight, followed by incubation with Protein A/G magnetic beads (HY-K0202, MedChemExpress) at 4 °C. The prepared immune complexes were analyzed by SDS-PAGE and Western blot.

#### **Dual-luciferase reporter assay**

The dual-luciferase reporter assay was used to assess CREB3L1’s transcriptional activation of the COL11A1 promoter. Cells were co-transfected with COL11A1 (pcDNA3.1) and CREB3L1 (pGL3) plasmids. Luciferase activity was measured 48 h post-transfection using the Dual-Luciferase Reporter Assay System.

### Antibodies, inhibitors and recombinant proteins

Antibodies used in this study: LAMC2 (1:200, 19698-1-AP, Proteintech, China), Pan Cytokeratin(1:200, 26411-1-AP, Proteintech, China), αSMA (1:10,000, 80008-1-RR, Proteintech), FAP (1:1000, ab53066, Abcam), ITGB1 (1:500, sc-374429, Santa Cruz Biotechnology), FOXP3 (1:1000, ab22510, Abcam), COL11 A1 (1:1000, GTX55142, GeneTex), GAPDH (1:10,000, R380626, Zen-Bioscience), CD44 (1:1000, ET1609-74, HUABIO), CREB3L1 (1:1000, MABE1151, Millipore), SMAD2/3 (1:1000, 5678, Cell Signaling Technology), p-SMAD2/3 (1:1000, 8828, Cell Signaling Technology), SMAD7 (1:1000, 25840-1-AP, Proteintech), Flag (1:1000, 14793, Cell Signaling Technology), TGF-β1 (1:1000, HA721143, HUABIO), PLAU (1:2000, 17968-1-AP, Proteintech), uPAR (1:1000, 10286-1-AP, Proteintech), STAT1 (1:5000, 10144-2-AP, Proteintech), IRF9 (1:1000, 14167-1-AP, Proteintech), GST (1:1000, sc-2027, Santa Cruz Biotechnology), IgG (1:100, 8828, Cell Signaling Technology), pY397-FAK (1:100, 3283, Cell Signaling Technology), FAK (1:100, 3285, Cell Signaling Technology), TGFβRI (1:100, ab31013, Abcam), TGFβRII (1:100, ab184948, Abcam), phospho-tyrosine (1:100, 9411, Cell Signaling Technology), HRP Conjugated Goat anti-Rabbit IgG polyclonal Antibody (1:20,000, HA1001, HUABIO), HRP Conjugated Goat anti-Mouse IgG polyclonal Antibody (1:20,000, HA1006, HUABIO). Small molecule inhibitors used in this study: SB431542 (HY-10431, MedChemExpress), PF429242 (SML0667, Sigma), VS-4718 (HY-13917, MedChemExpress). Recombinant proteins used in this study: human PLAU (HY-P71050, MedChemExpress), human TGF-β1 (HY-P7118, MedChemExpress). Protease inhibitor cocktail (HY-K0010, MedChemExpress) was used for protein extraction.

### scRNA-seq data preprocessing and quality control

We have established a precancerous-malignant transition scRNA-seq cohort, incorporating 9 samples from HRA004648 (Patient2, Patient10, Patient13) [5]. Our cohort encompasses normal tissue, precancerous tissue, and cancerous tissue from the same patients. The raw data was processed using “CellRanger” (version 3.1.0) with default parameters and mapped to human GRCh38 (version32) to generate gene count matrices by barcoded reads per cell for each sample. The “Seurat” R package (Version 5.1.0) was utilized for quality control and analysis. In scRNA-seq data quality control, single cells with 200–6000 genes and a mitochondrial UMI percentage <10% were retained. Raw UMI counts were normalized using the SCTransform function. Possible batch effects from patient-specific expression patterns were adjusted using the “Harmony” R package (version 1.2.1). Dimension reduction was performed using principal-component analysis (PCA) with the “RunPCA” function, and unsupervised clustering was conducted with the “FindNeighbors” function. We employed the “FindClusters” function to identify cell clusters across different resolutions and reduced data dimensionality using t-SNE in the “RunTSNE” function.

Based on cell clusters from unsupervised clustering, the “FindAllMarkers” function was used to identify differentially expressed genes (DEGs) across clusters. DEGs were filtered using criteria of log2FC > 1 and adjusted *p* value < 0.05, with only upregulated DEGs considered. Major cell types were assigned to cell clusters based on the normalized expression of known marker genes in previous study. Differential cell abundance analysis of major cell types was performed using the MiloR R package (Version 2.2.0).

### Visium spatial transcriptomics analysis

We have assembled a precancerous-malignant transition spatial transcriptomics (VISIUM) cohort, with 16 samples from GSE208253 and GSE220978 [38, 39]. Our cohort encompasses the malignant progression process of oral squamous cell carcinoma. The spatial transcriptomics data from all samples had histopathological examination and sufficient high-quality data. The VISIUM cohort was imported into the R package “Seurat” (Version 5.1.0). Data normalization was performed using the “SCTransform” function, followed by dimensionality reduction using the RunPCA function. Unsupervised clustering and visualization were executed with the “FindNeighbors”, “FindClusters”, and “RunUMAP” functions. The spatial expression patterns of genes and gene sets were visualized using the “SpatialFeaturePlot” function. The scRNA-seq cohort was used as reference data and integrated into the VISIUM cohort via “spacexr” R package (Version 2.0.0) to perform spatial mapping and decompose the cell type proportions in each spot. The cell type proportions in each spot were obtained using the “run.RCTD” function, and the final cellular identity was determined based on the first type.

### Expression programs of epithelial cells heterogeneity

Non-negative matrix factorization (NMF) analysis was applied to the epithelial cells in the scRNA-seq cohort using the “GeneNMF” R package (Version 0.6.0). After selecting the 2000 most highly expressed genes, the centered expression matrix was adjusted by setting negative values to zero. NMF runs were conducted with various K values (k = 4–9). We further filtered out meta-programs containing fewer than 10 genes or having a sample coverage rate below 50%, resulting in the identification of six meta-programs. To ensure the reliability and measurability of the gene sets for each meta-program across all samples, we assessed the coverage rate of genes from each meta-program in both the scRNA-seq cohort and VISIUM cohort. On average, over 80% of the genes for each meta-program were consistently identified across all samples. The signature scores were calculated using the “AddModuleScore” function in the R package “Seurat” (version 5.1.0). Protein-protein interaction (PPI) networks for the meta-program gene sets were constructed using the STRING database (version 12.0), and proteins that could not form network connections were excluded.

### Pseudotime analysis

To elucidate the differentiation trajectories of various cell types and the dynamic changes in gene expression during the malignant progression of oral squamous cell carcinoma, we performed trajectory analysis using the R package “Monocle2” (Version 2.32.0). We created a new CellDataSet object by extracting count matrices and metadata from the Seurat object. After identifying highly variable genes between clusters with the “differentialGeneTest” function, we performed dimensionality reduction using the “DDRTree” algorithm. The “plot_pseudotime_heatmap” function visualizes gene expression changes along the pseudotime trajectory through a heatmap. Finally, we established branched expression analysis modeling (BEAM) based on trajectory characteristics to analyze each branch’s cell fates and transcriptomic patterns using the “plot_genes_branched_pseudotime” function.

### Cell communication analysis

The R package Liana (Version 0.1.14) was used to infer communication between epithelial cell and other cell types, Liana has provided a framework that integrates numerous algorithms and database resources for inferring cell—cell communication. We constructed a Liana object using the “liana_wrap” function with default parameters. The Connectome output was filtered to retain only interactome links with *P* values < 0.05 for both ligand and receptor. Finally, the “liana_dotplot” function was used to visualize ligand-receptor pairs. The R package ggplot2 (Version 3.5.1) was employed to present the spatial expression of these interactions.

### Gene set variation analysis

To gain functional and mechanistic insights into cell clusters or gene modules, gene ontology and KEGG pathway enrichment analyses were performed using the R package “clusterProfiler” (Version.4.12.2), with gene pathways having an adjusted *p* value < 0.05 considered significantly enriched. Pathway enrichment analysis was conducted using the “GSVA” R package (Version 1.52.3) with selected Hallmark pathways from the MSigDB database (version 7.1). Additionally, CNVs in epithelial cells were estimated using InferCNV (version 1.20).

### Defining the tumor and TME components

We quantified tumor cell purity in each spatial transcriptomics subspot using the “ESTIMATE” R package (version 1.0.13), a widely recognized tool for measuring stromal, immune and cancer cell levels in bulk tumor RNA-seq data. Given the subcellular-level resolution of our visium cohort, each spot is treated as a bulk RNA-seq sample containing multiple cells, allowing the application of same deconvolution methods. We extracted each subspot count matrices from the Seurat object to generate a GCT format file. Then, we used the “estimateScore” function to calculate stromal, immune, and ESTIMATE scores for each spot. We used K-means clustering to create five clusters in each spatial transcriptomics sample. The 4 and 5 clusters, which had the highest ESTIMATE scores, corresponded to the lowest tumor cell purity and were defined as adjacent stroma (AS). Conversely, the 1 and 2 clusters, with the lowest ESTIMATE scores, showed the highest tumor cell purity and were designated as the tumor center (TC). The 3 cluster was defined as the invasive front (IF) [40–42]. The accuracy of these designations was validated based on H&E-stained tissue sections and p-EMT scores.

### Dissecting spatial autocorrelation

To evaluate the spatial autocorrelation of pathway activity, ligand-receptor pairs, and gene sets in spatial transcriptomics, we applied the “MISTy” R package (version 1.10.0) to our VISIUM cohort. Using the “Seurat” R package (Version 5.1.0), we obtained spatial coordinates for the lowest (clusters 4 & 5) and highest (clusters 1 & 2) tumor cell purity via the “GetTissueCoordinates” function. The radius was determined as the average distance to the nearest point plus one standard deviation. We then used the “create_initial_view” function to incorporate variables and collected results with the “collect_results” function. A higher aggregated importance value signifies stronger spatial autocorrelation.

To explore the crosstalk between epithelial cells secreting PLAU and the subtype conversion of fibroblast, we first determined the final cellular identity of each spot based on the deconvolution results from the “spacexr” R package. We then calculated the expression levels of COL11A1^+^ Fib and PLAU across all spatial transcriptomics spots and used spatial coordinates to compute the Euclidean distance from each tumor spot to its nearest neighbor. These distances were utilized in subsequent analyses. Epithelial cell spots with higher PLAU expression were more likely to induce the conversion of fibroblast to the COL11A1^+^ Fib.

### Deconvolution

To deeply explore the precancerous-malignant transition tumor microenvironment in TCGA-HNSCC, we utilized the “BayesPrism” R package (Version 2.2.2). We integrated scRNA-seq data with TCGA-HNSCC data, filtering out genes related to actin, ribosomal genes, and those on the X or Y chromosomes. By simulating cell states and characteristics, we inferred the specific gene expression of cell types and their proportions within the TCGA-HNSCC samples. We also employed the “spacexr” R package (Version 2.2.0) to deconvolve spatial transcriptomics (ST) data and determine cell type composition in each spot. SpaceX references annotated scRNA-seq data to generate gene expression profiles for each cell type and fits a probabilistic model to estimate the proportion of each cell type. The cellular makeup of each spots were obtained using the “run.RCTD” function, and the final cellular identity was determined based on the first type.

### Survival analysis

To evaluate the prognostic performance of the COL11A1^+^ Fib signature, bulk RNA-seq data from the GSE41613, GSE42743 and TCGA databases were utilized. Patients were categorized into high- and low-risk groups based on the median score of the COL11A1^+^ Fib signature, which comprises 30 genes. Kaplan-Meier survival analyses were performed using the “survminer” and “survival” R packages (Version 3.8.3).

### Transcription factor regulon analysis

The gene regulatory networks (GRNs) in scRNA-seq data were constructed using the SCENIC workflow (version 1.2.4). Transcription factor-binding motif analysis was performed using RcisTarget (version 3.2.2) based on the hg19 reference genome database. Normalized enrichment scores (NESs) were calculated for each transcription factor-binding motif.

### Statistical analysis

Statistical analyses were carried out with R software (version 4.4.1). Two-group comparisons were assessed using independent-samples *t* tests, and multi-group comparisons were evaluated by one-way ANOVA. A threshold of *p* ≤ 0.05 was adopted to determine statistical significance. Parametric tests were applied to datasets meeting normality assumptions; otherwise, non-parametric methods were used. For flow cytometry, data acquisition and compensation were performed in BD FACS-Diva, followed by export as FCS 3.1 files and subsequent analysis using FlowJo (version 10.8.1).

## Supplementary information

BioRender license agreement

Supporting Information

Supplement Table 1

Supplement Table 2

Supplement Table 3

Supplement Table 4

Original blots

## Data Availability

The raw and processed data generated in this study were deposited in the Genome Sequence Archive (GSA) in the BIG Data Center (https://ngdc.cncb.ac.cn/gsa-human/), Beijing Institute of Genomics (BIG), Chinese Academy of Sciences, under accession number OMIX012780. The processed scRNA-seq, RNA-seq and ST data of OSCC used in the study are obtained from: HRA004648, EGAS00001004809, GSE236581 (scRNA-seq) [5, 43, 44]. GSE208253, GSE220978, HRA002509 (ST-seq) [38, 39, 45]. GSE41613, GSE42743, GSE37991, GSE184616, TCGA-HNSCC (https://portal.gdc.cancer.gov/) (RNA-seq) [46–48]. Source data are provided with this paper. Codes were implemented in R 4.4.1 and are deposited in https://github.com/XiongYili011006/OSCC.
