# Supplementary material for: The PLAU-ITGB1-TGF-β axis orchestrates spatiotemporal reprogramming of fibroblasts at the invasive front to drive dual immunosuppressive niche and immunotherapy resistance in oral squamous cell carcinoma
Source: Cell Death Dis. 2026 Jul 16;17(1):784. doi: 10.1038/s41419-026-09110-4 (PMC13558778; doi:10.1038/s41419-026-09110-4)
Supplement: Supplementary file 2 [file 41419_2026_9110_MOESM7_ESM.pdf]

Fig3-F

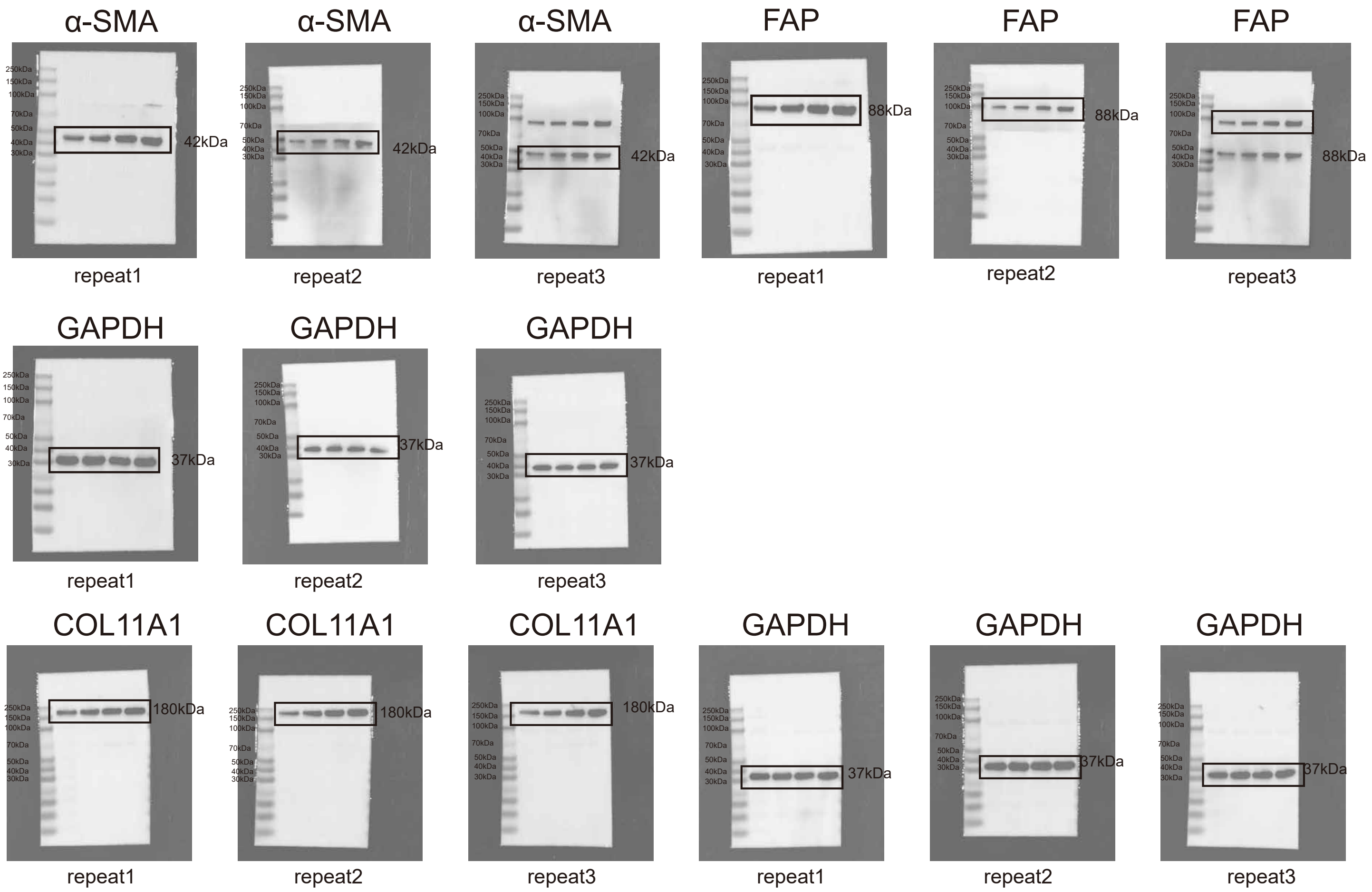

Fig3-G

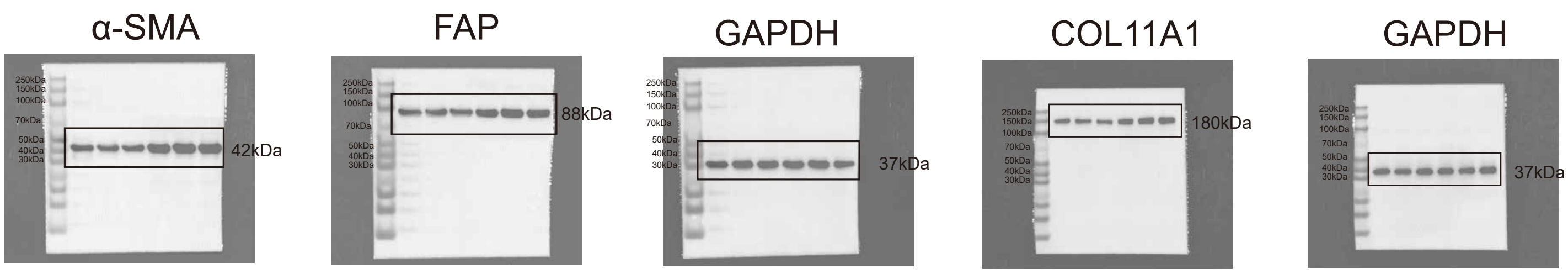

Fig3-H

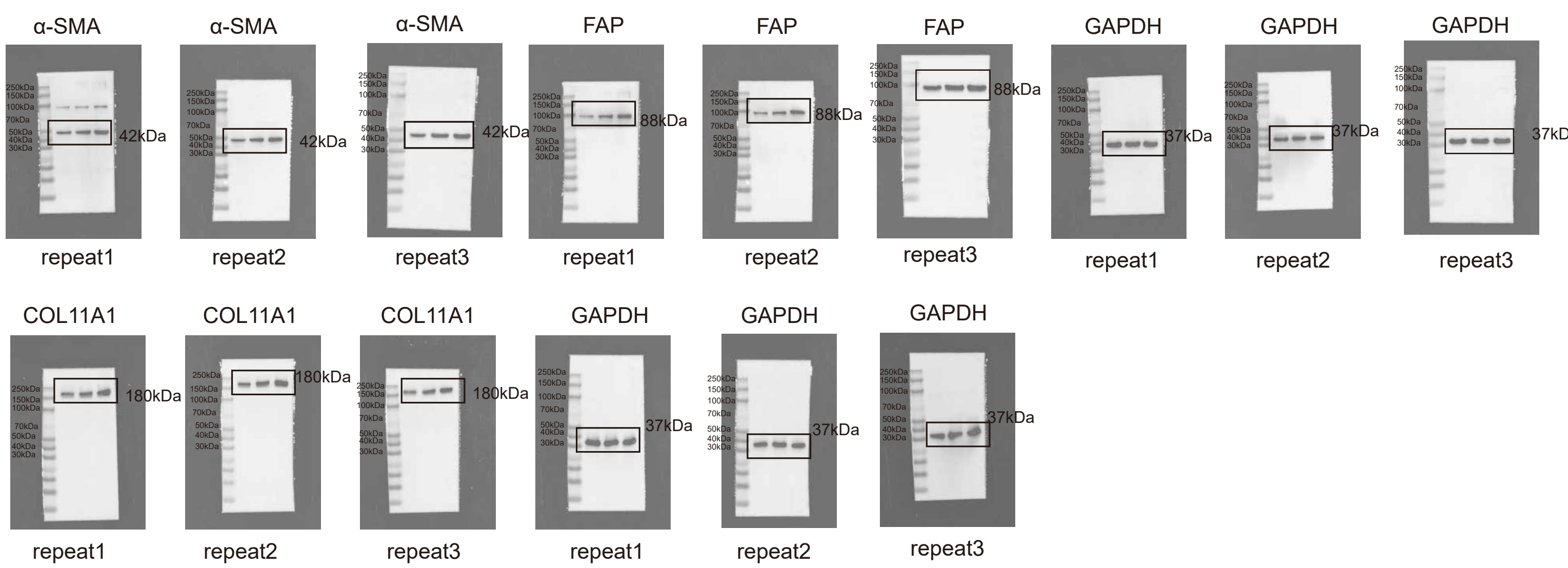

Supplement Fig3-J

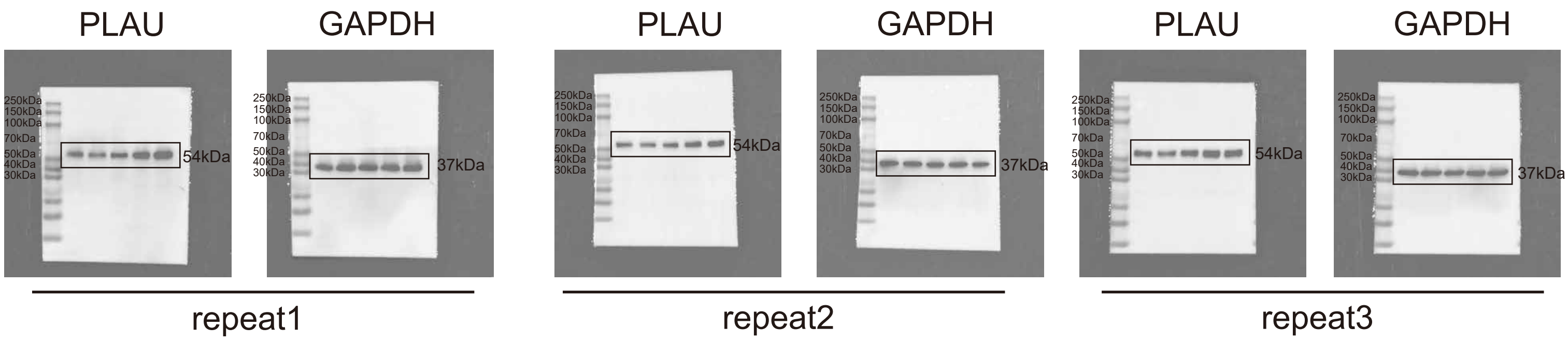

Supplement Fig3-K

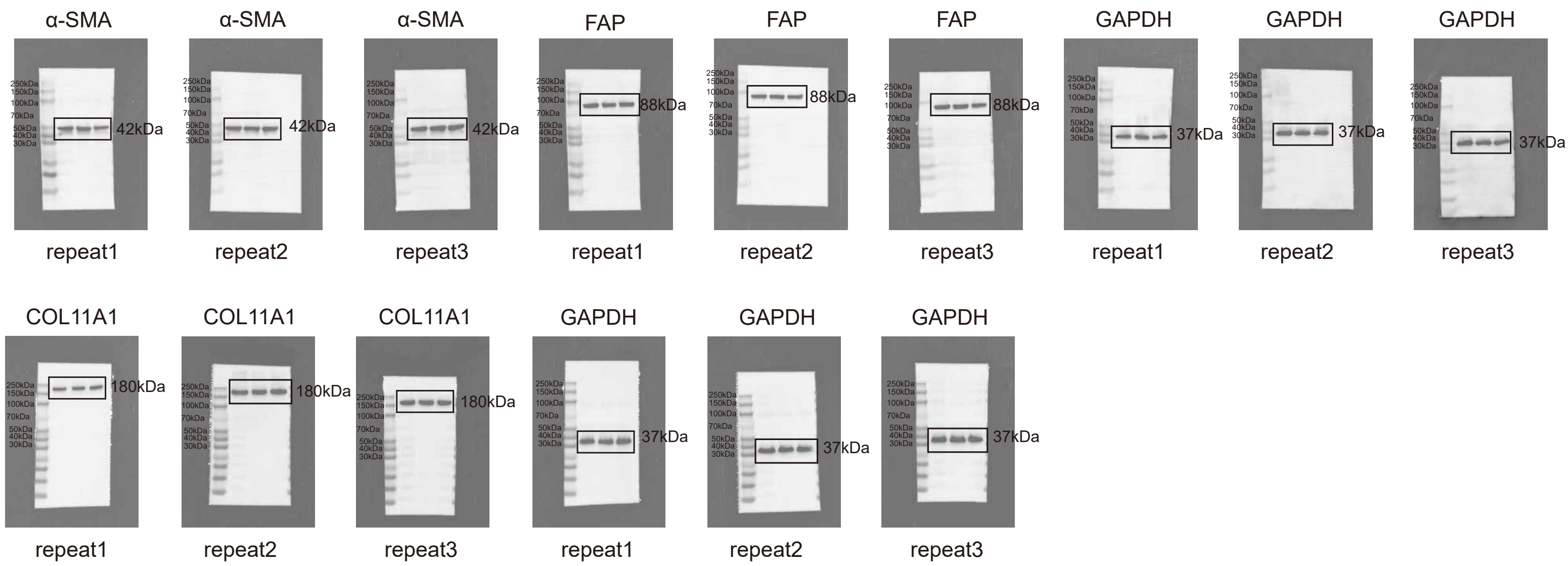

Supplement Fig3-L

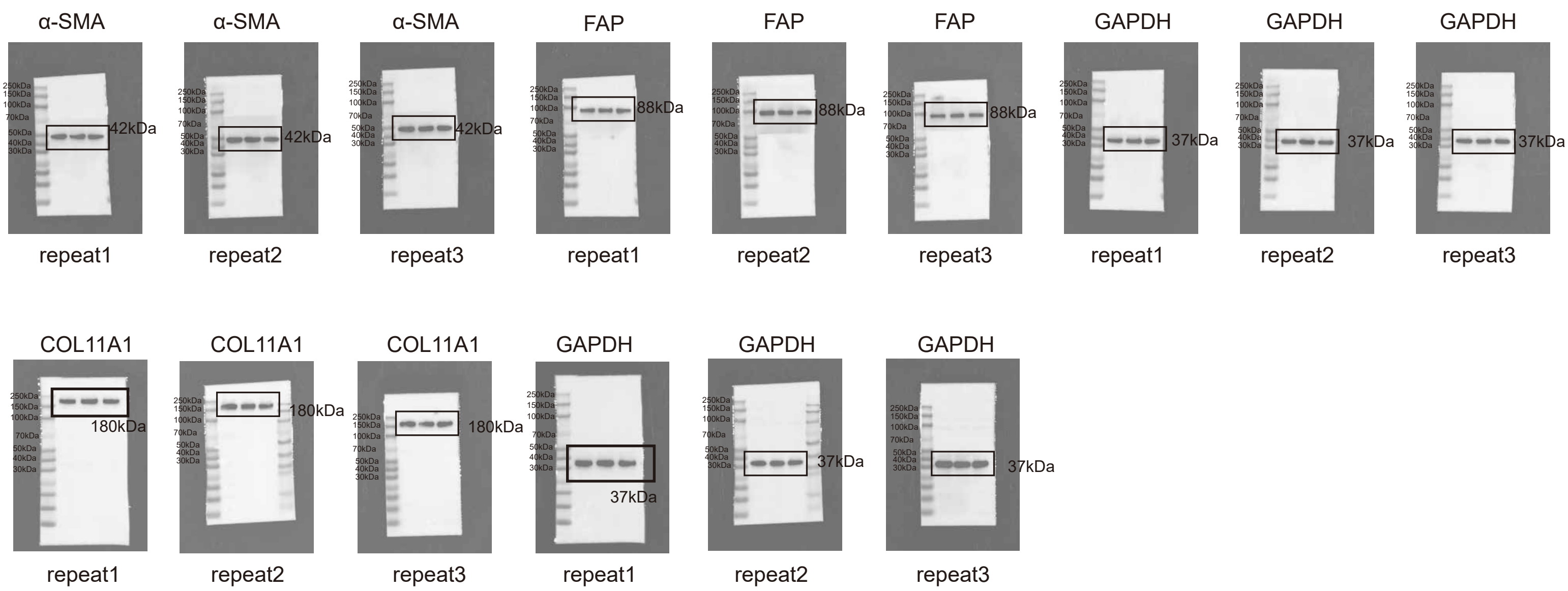

Supplement Fig3-M

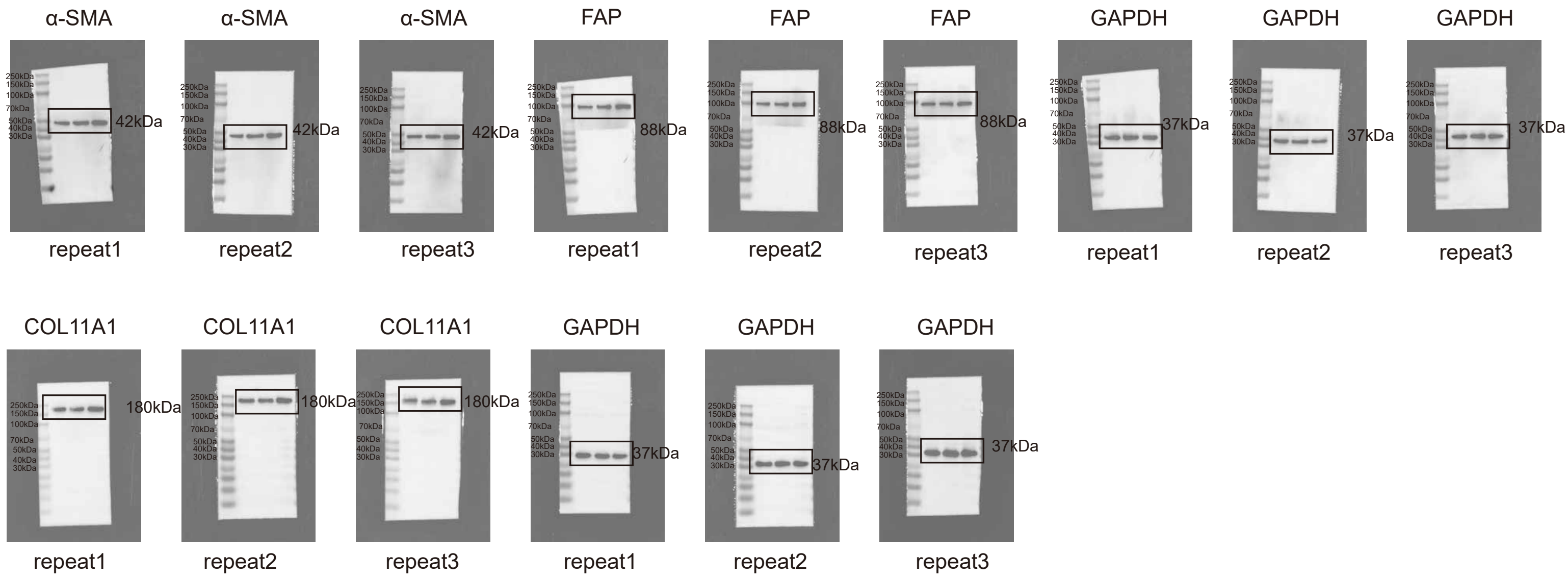

Supplement Fig3-N

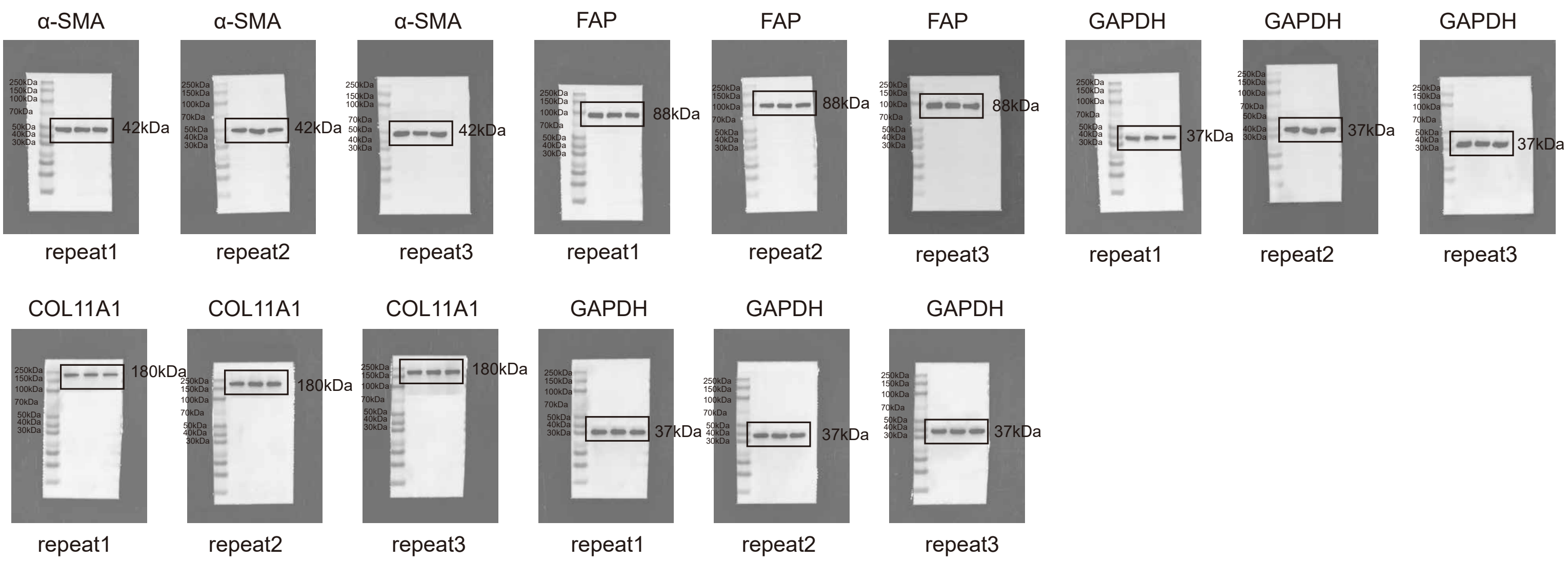

Supplement Fig3-O

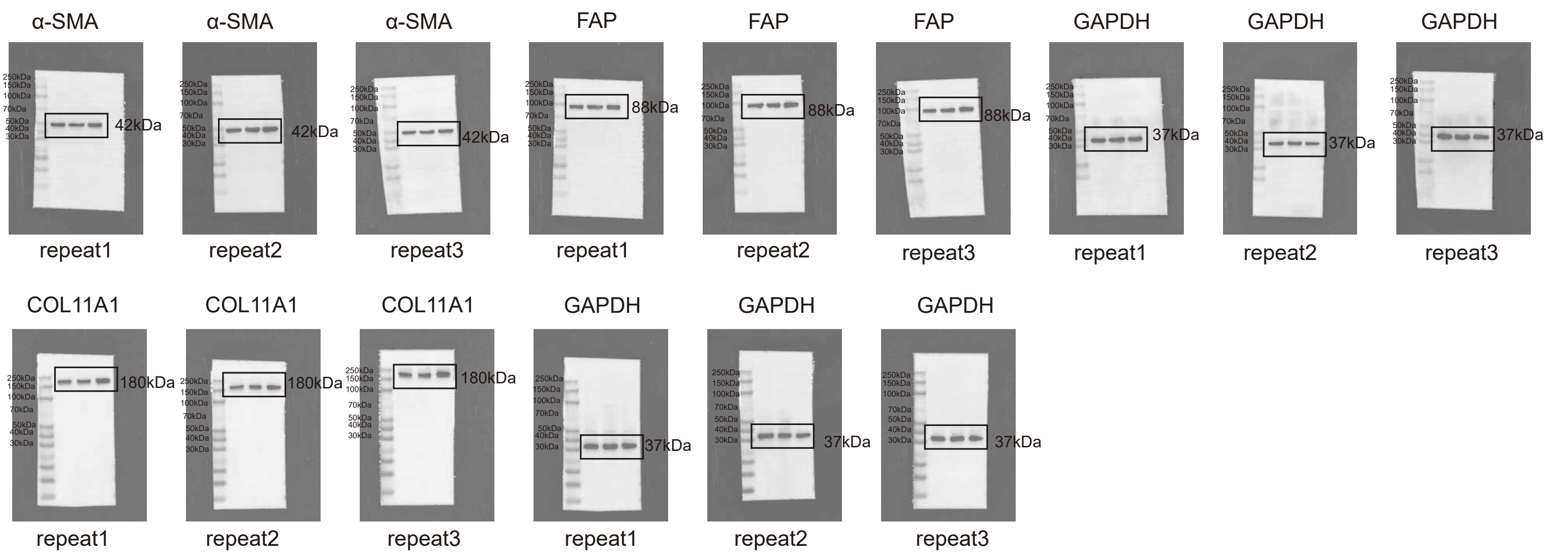

Fig4-C

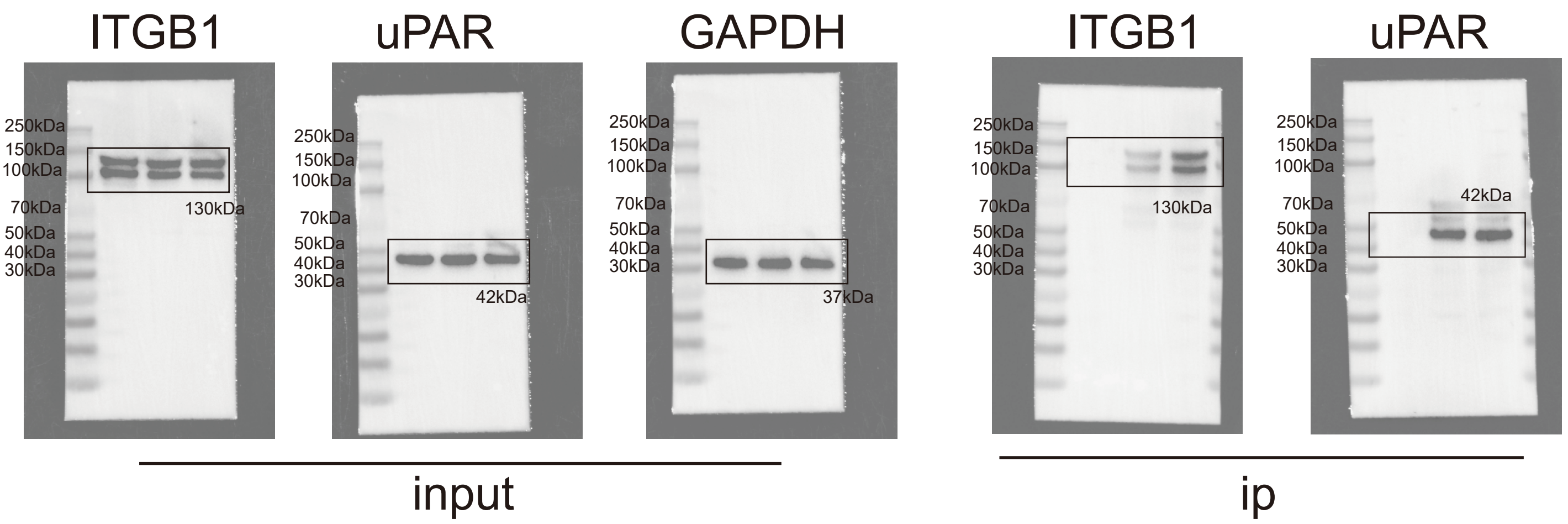

Fig4-H

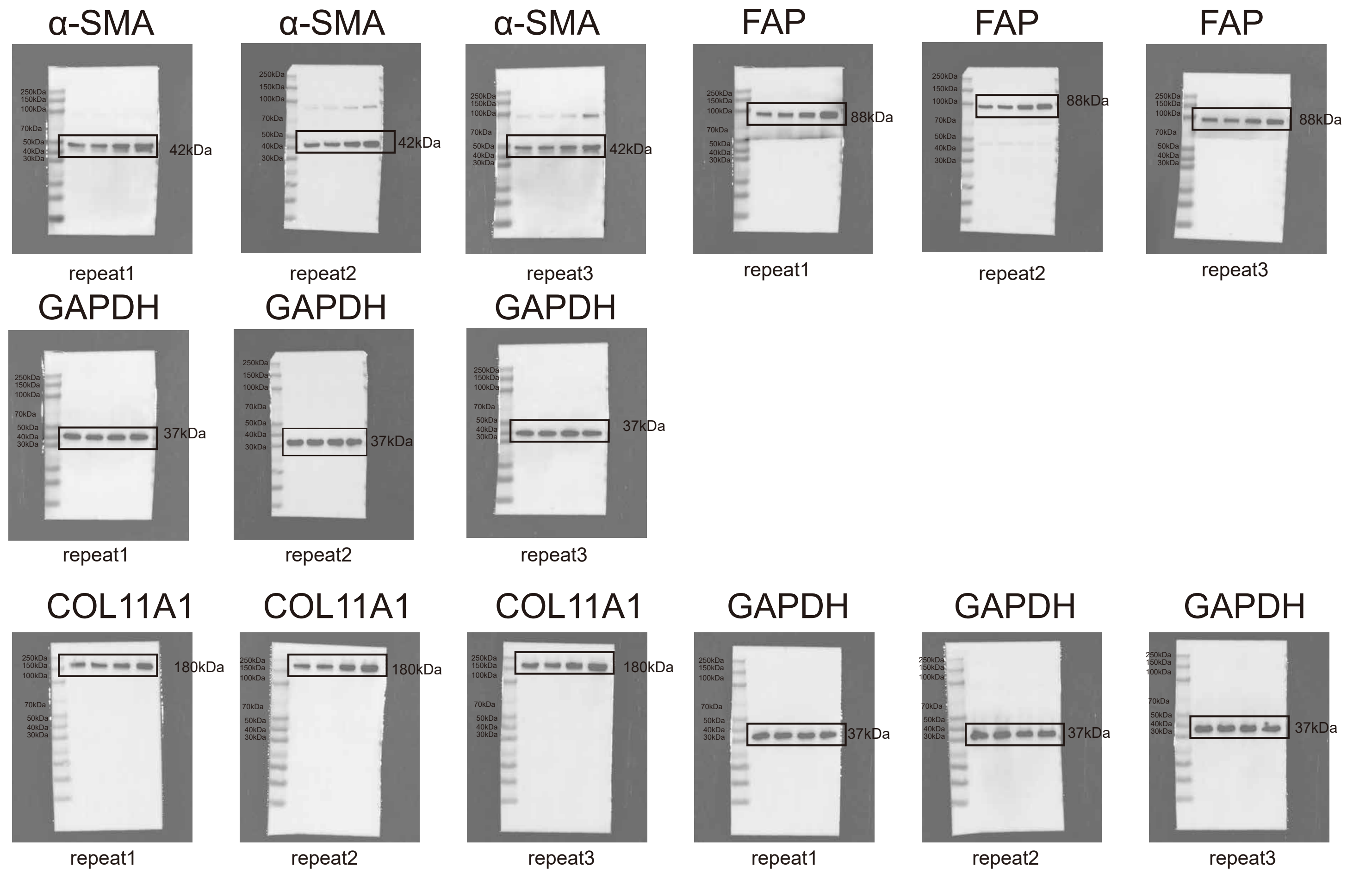

Supplement Fig4-E

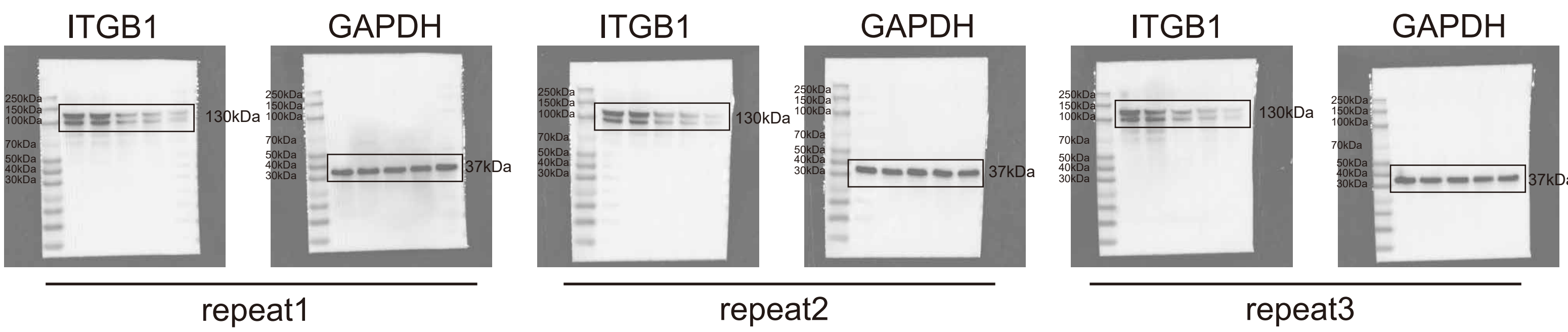

Fig5-F

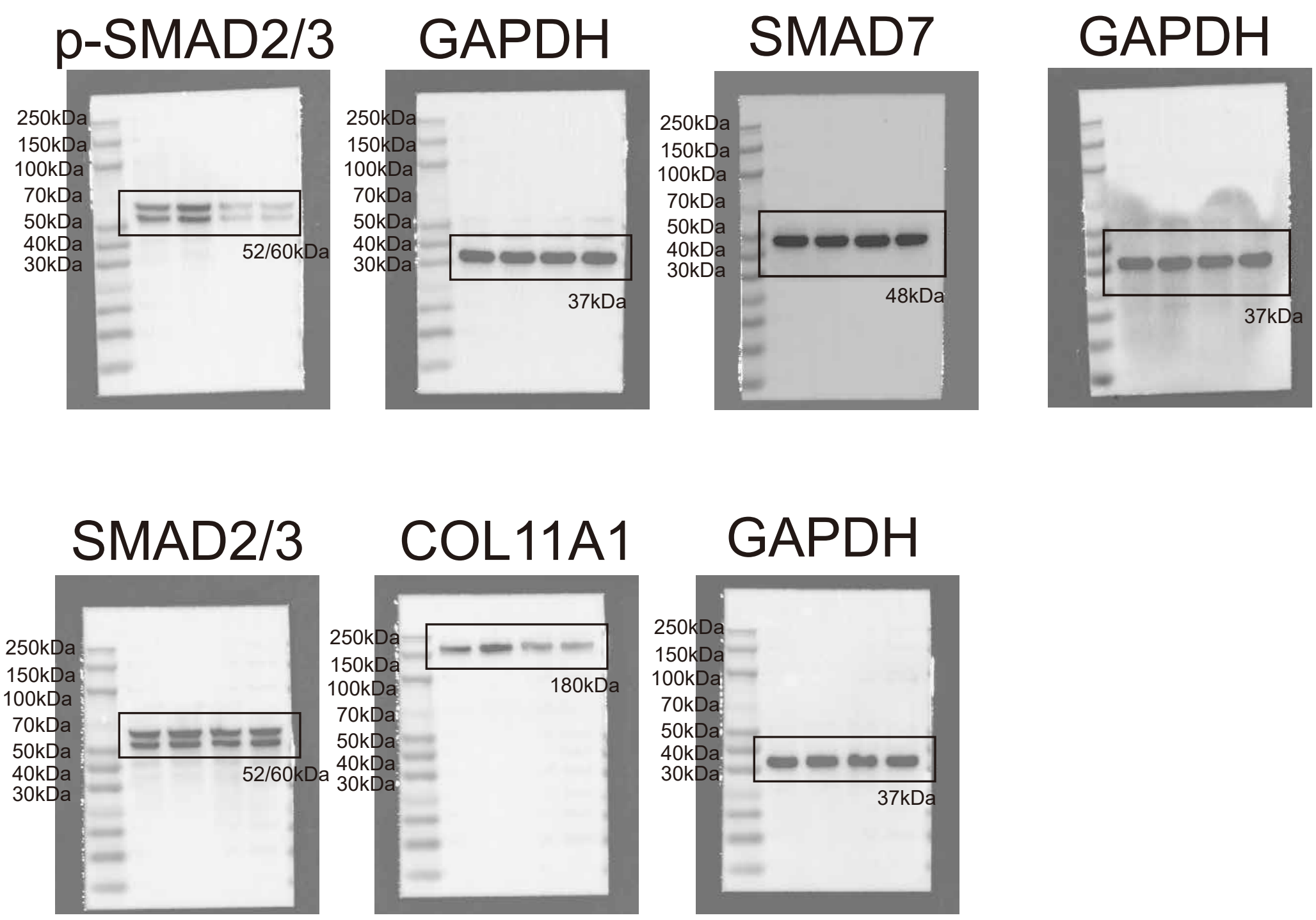

Fig5-G

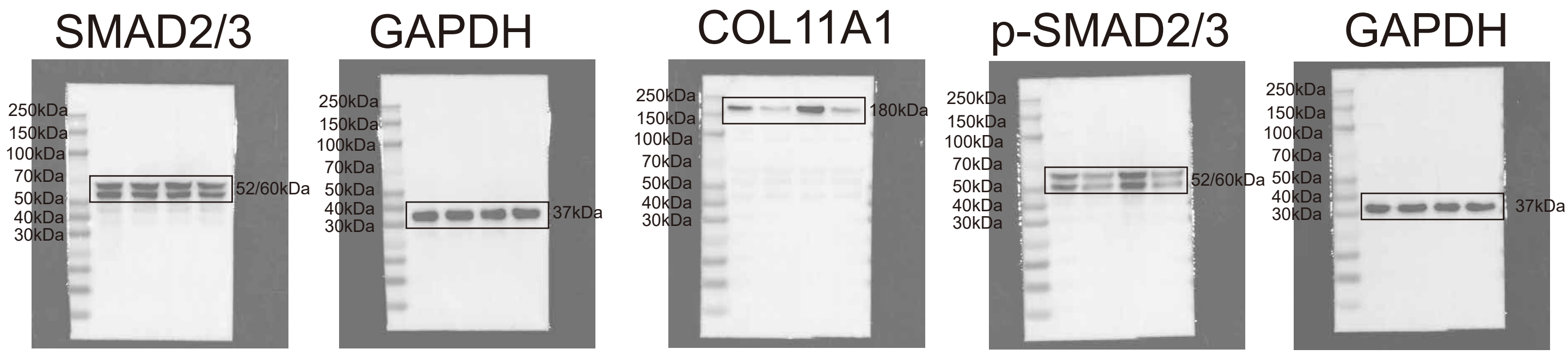

Fig5-H

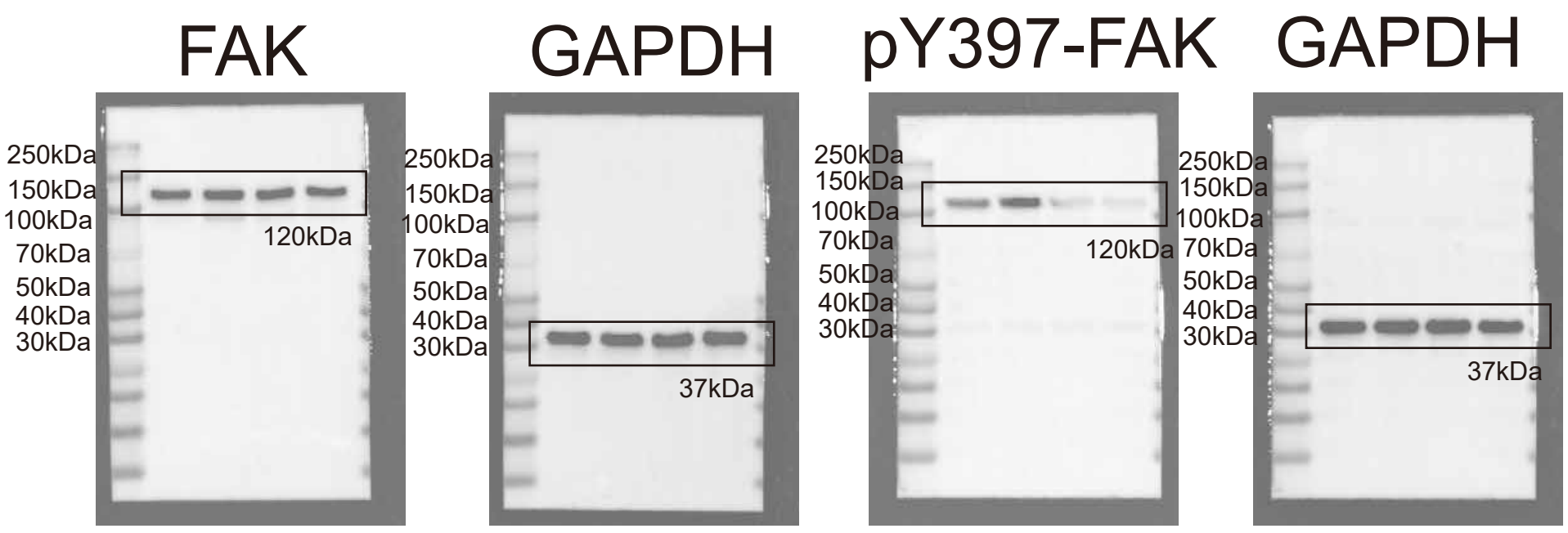

Fig5-I

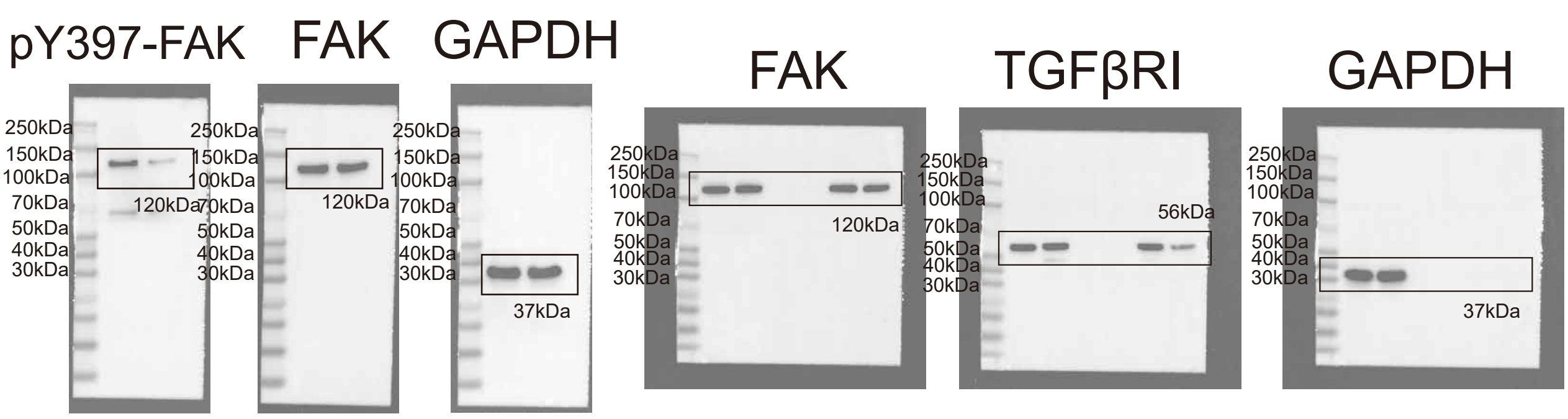

Fig5-J

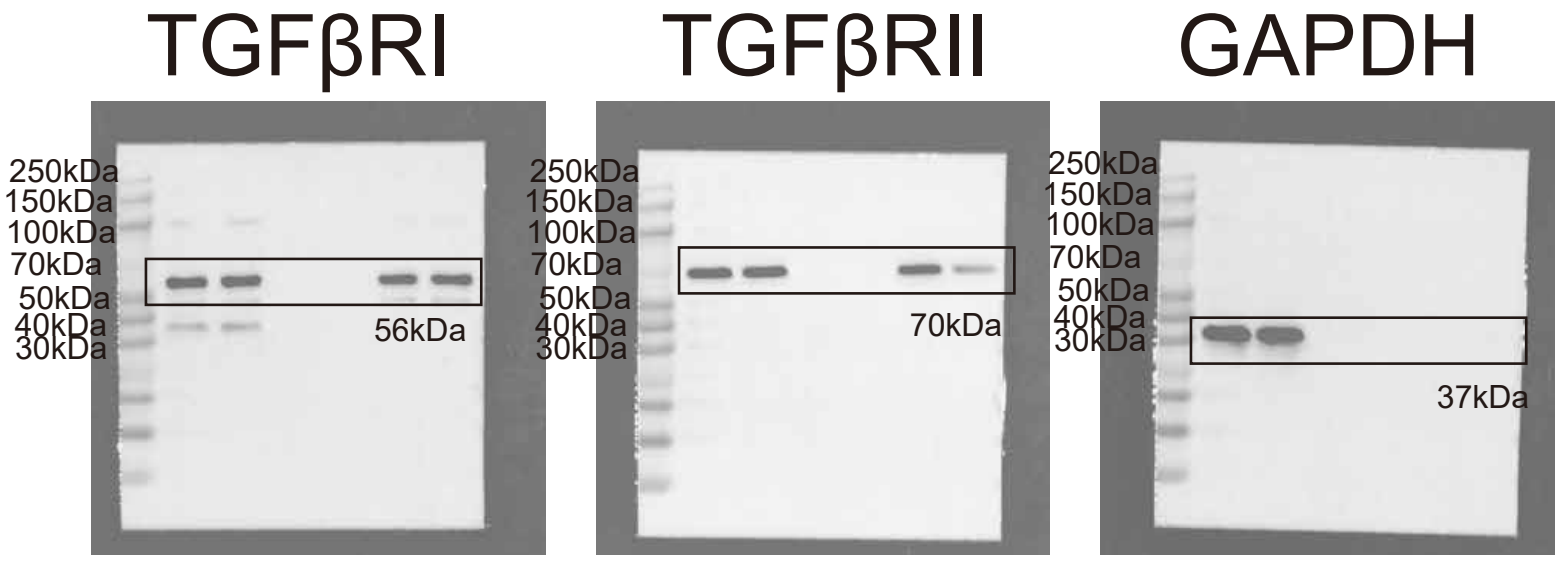

Supplement Fig5-A

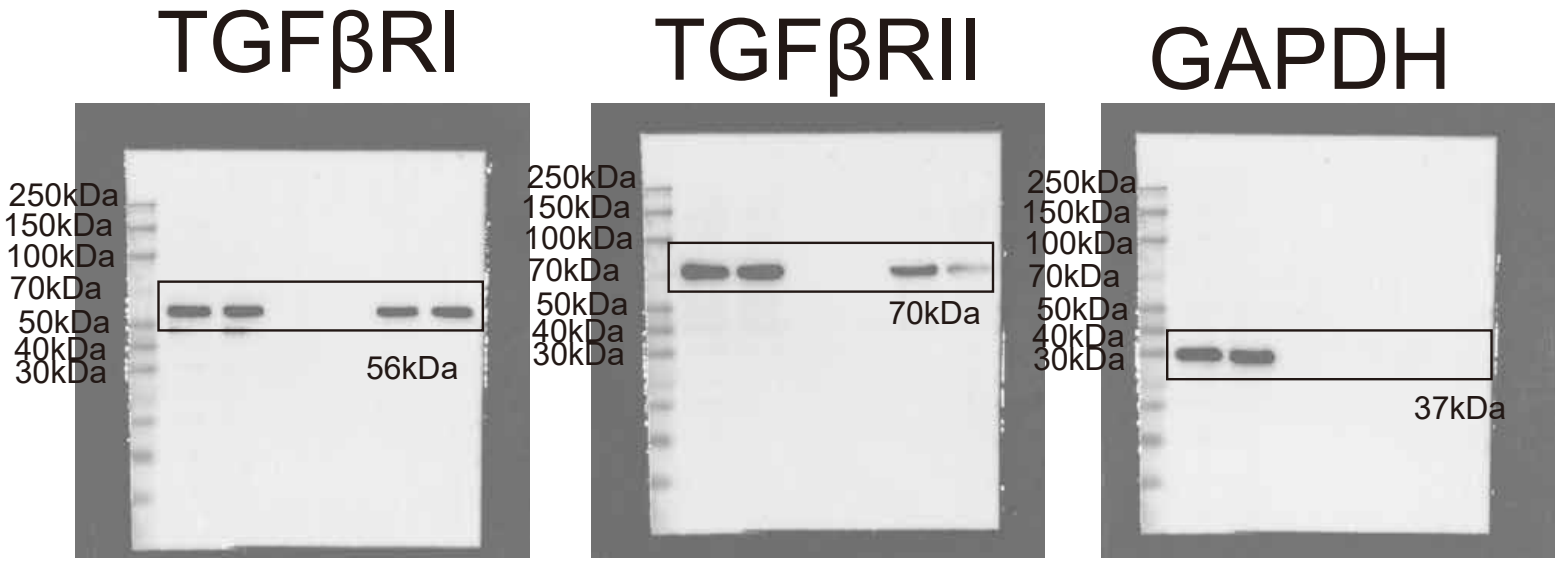

Supplement Fig5-B

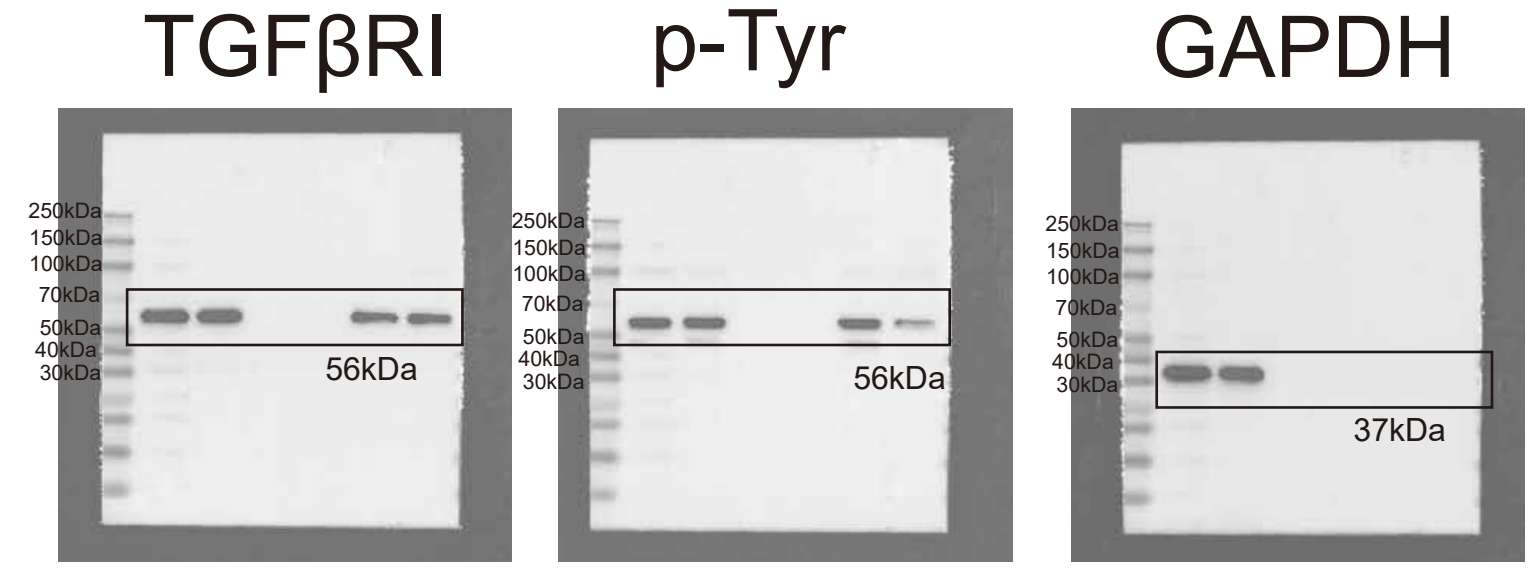

Fig6-F

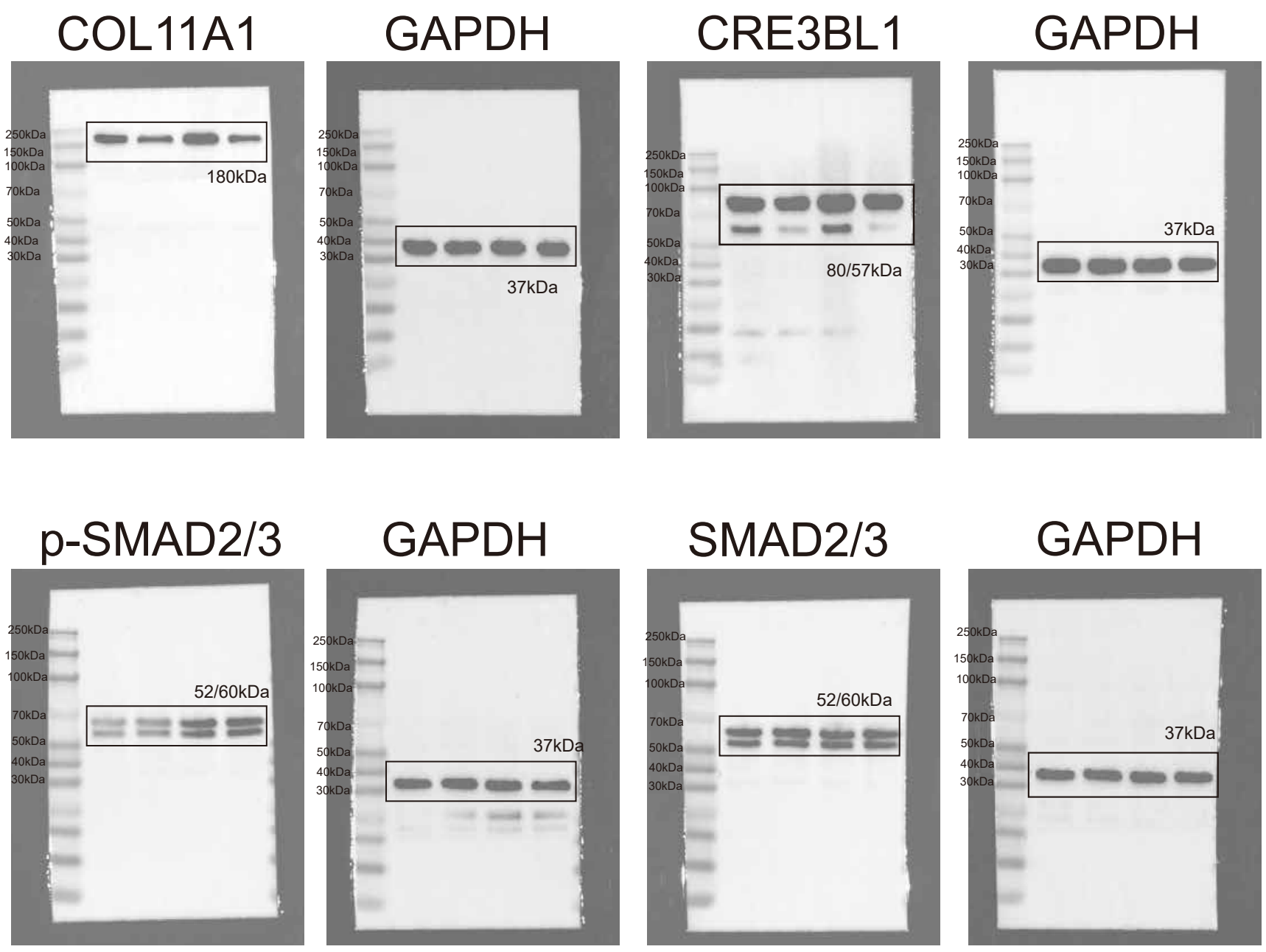

Fig6-I

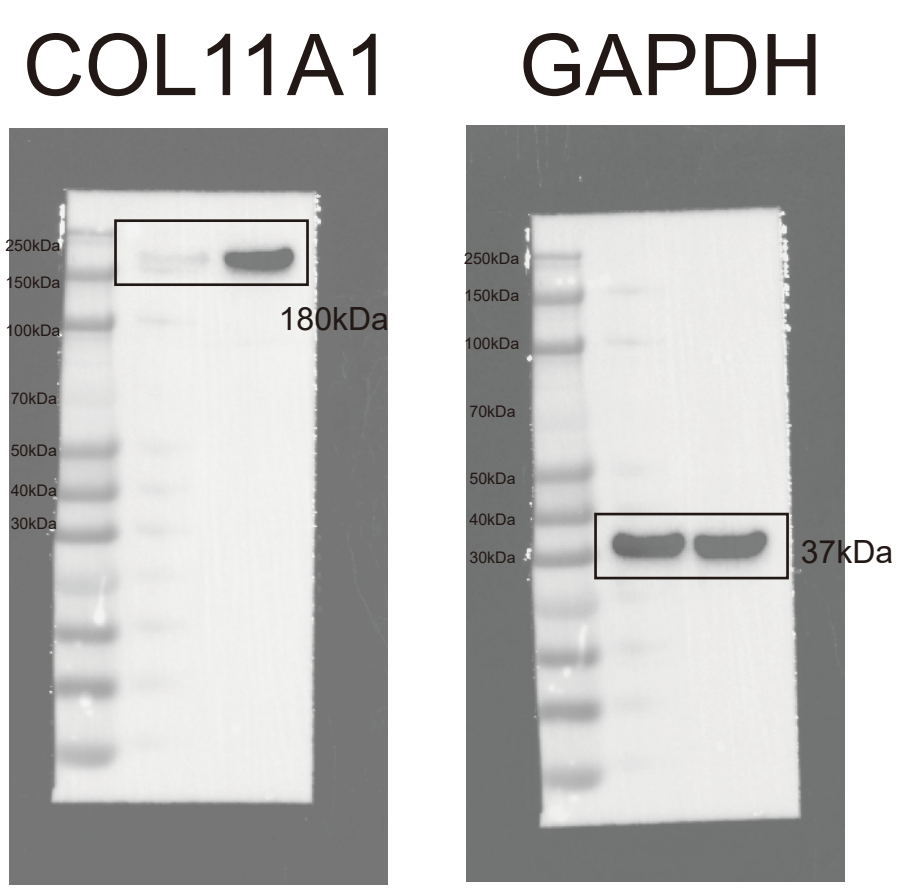

Fig6-K

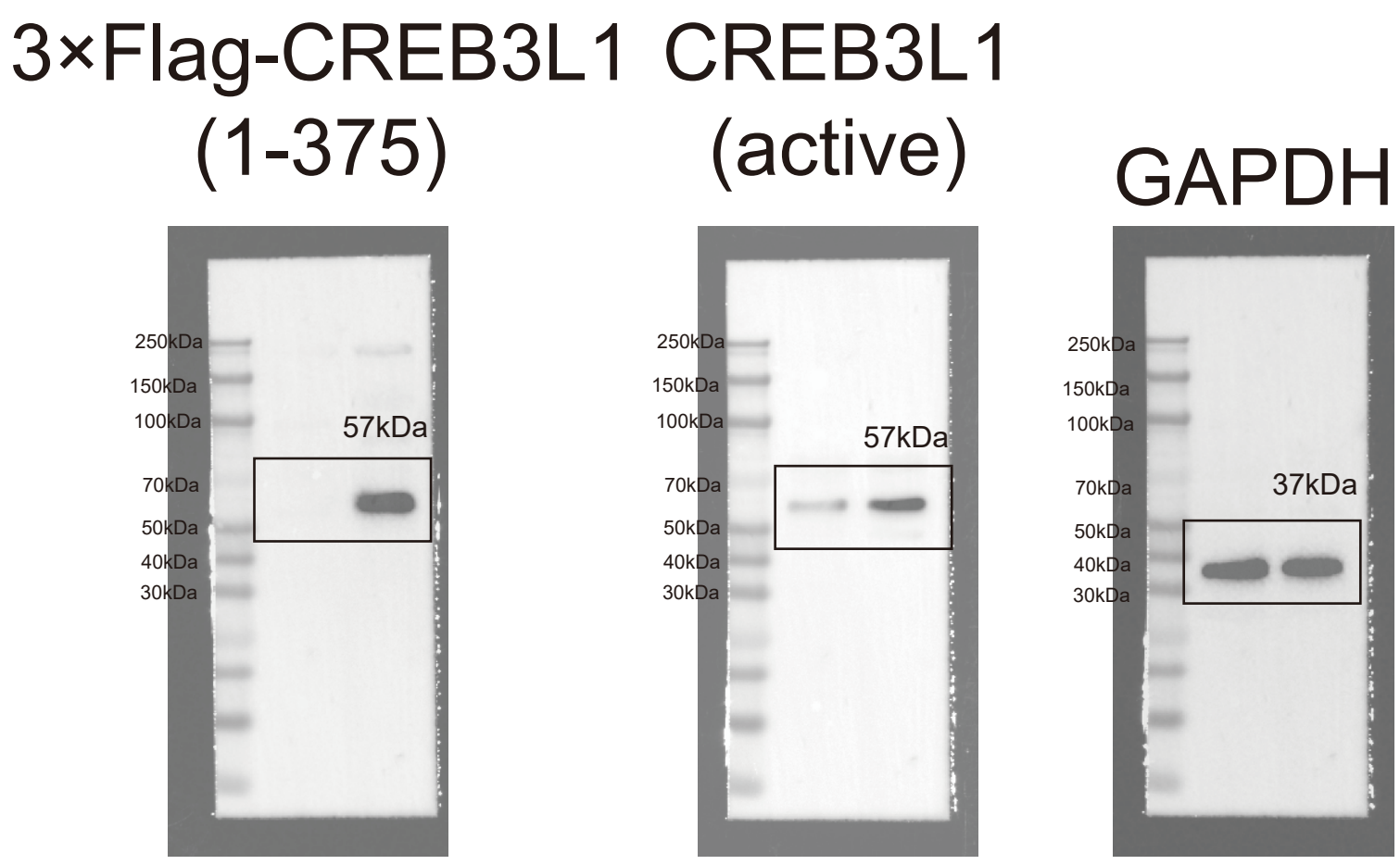

Supplement Fig6-C

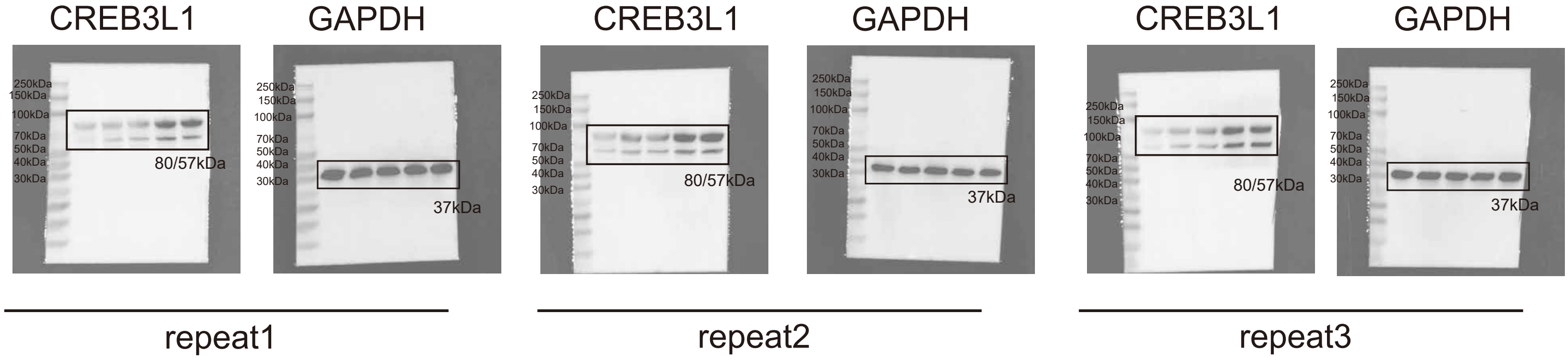

Supplement Fig6-D

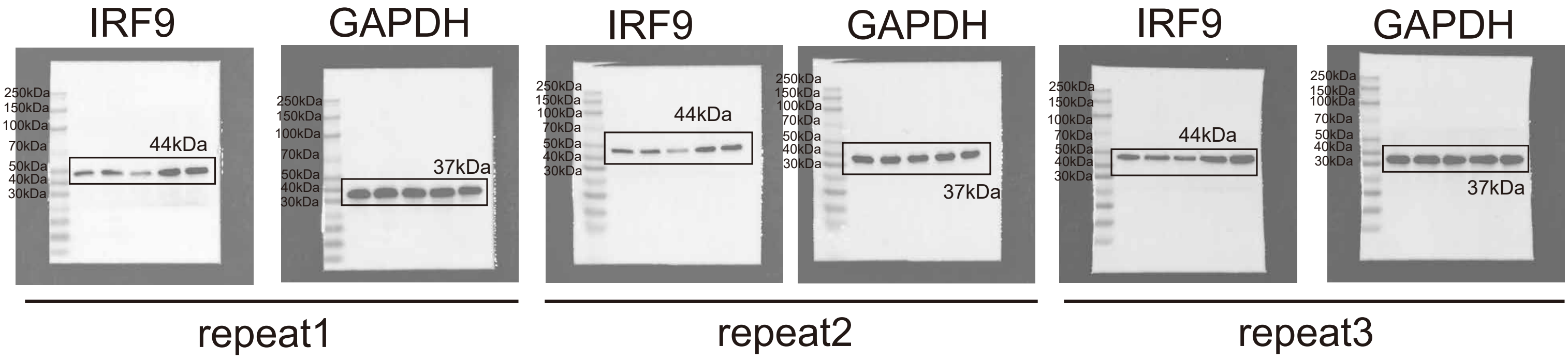

Supplement Fig6-E

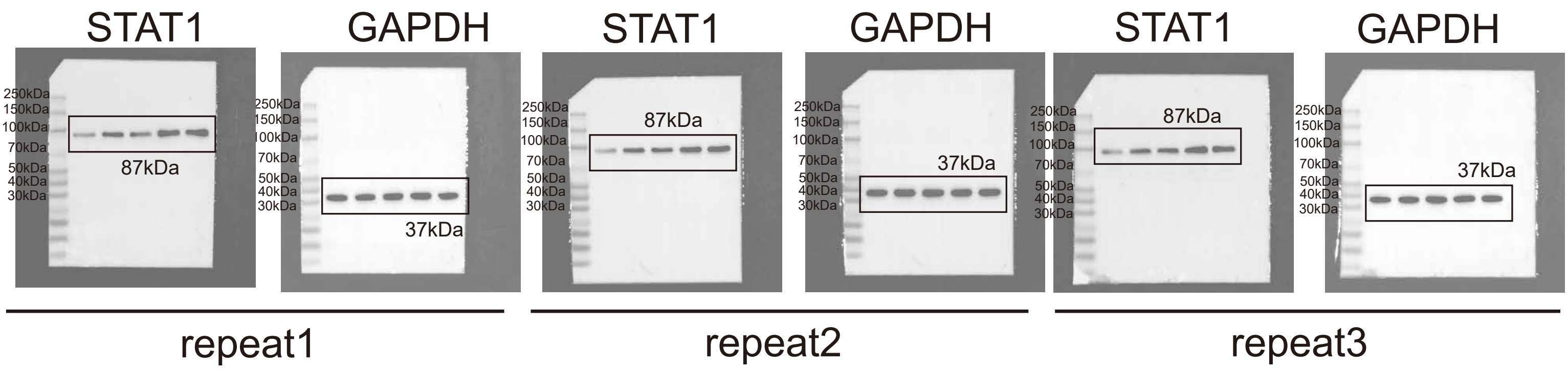

Fig7-R

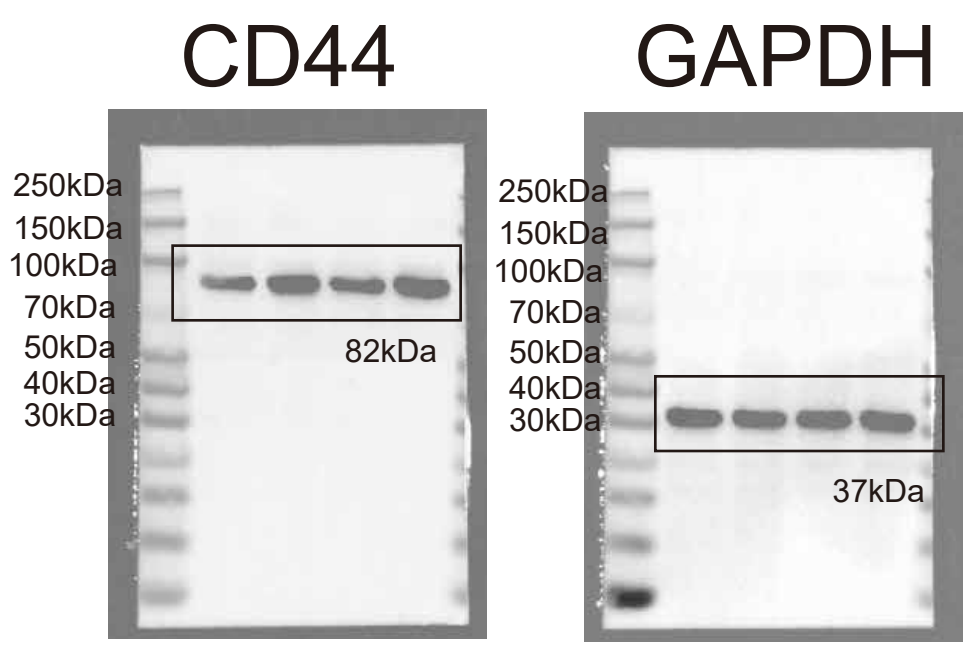

Supplement Fig7-D

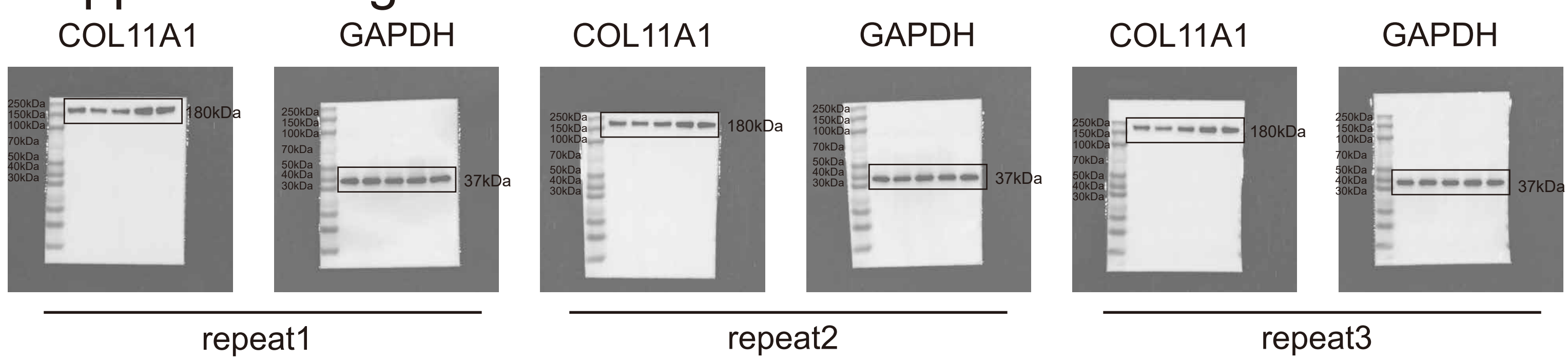

Fig8-R

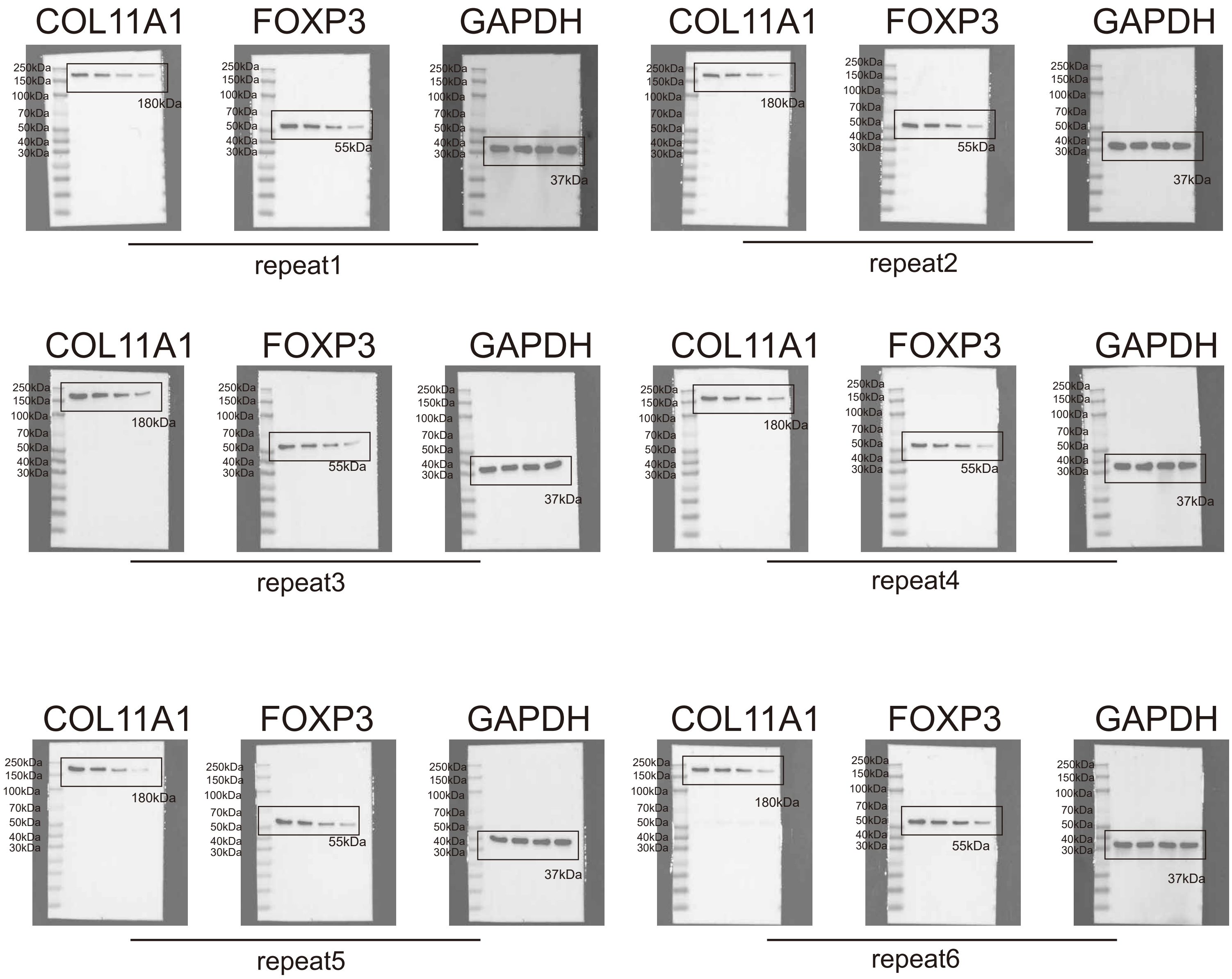
COL11A1
FOXP3
GAPDH
180kDa
55kDa
37kDa
repeat1
COL11A1
FOXP3
GAPDH
180kDa
55kDa
37kDa
repeat2
COL11A1
FOXP3
GAPDH
180kDa
55kDa
37kDa
repeat3
COL11A1
FOXP3
GAPDH
180kDa
55kDa
37kDa
repeat4
COL11A1
FOXP3
GAPDH
180kDa
55kDa
37kDa
repeat5
COL11A1
FOXP3
GAPDH
180kDa
55kDa
37kDa
repeat6
