# Supplementary material for: The PLAU-ITGB1-TGF-β axis orchestrates spatiotemporal reprogramming of fibroblasts at the invasive front to drive dual immunosuppressive niche and immunotherapy resistance in oral squamous cell carcinoma
Source: Cell Death Dis. 2026 Jul 16;17(1):784. doi: 10.1038/s41419-026-09110-4 (PMC13558778; doi:10.1038/s41419-026-09110-4)
Supplement: Supplementary file 3 [file 41419_2026_9110_MOESM2_ESM.doc]

**The PLAU-ITGB1-TGF-β axis orchestrates spatiotemporal reprogramming of fibroblasts at the invasive front to drive dual immunosuppressive niche and immunotherapy resistance in oral squamous cell carcinoma**

**Author:** Yili Xiong,^1,2,5^ Shaopeng Liu,^1,2,5^ Lianyu Zhao,^1,2,5^ Xinze Liu,^1,2^ Hanzhi Zhang,^1,2^ Haoran Wang,^1,2^ Dongsheng Zhang,^1,2,*^ Changlong Wang,^3,4,*^ Haiwei Wu,^1,2,*^

**Affiliations:**

^1^Department of Stomatology, Shandong Provincial Hospital Affiliated to Shandong First Medical University, Jinan, Shandong 250021, P.R. China

^2^School of Stomatology, Shandong First Medical University & Shandong Academy of Medical Sciences, Jinan, Shandong 250117, P.R. China

^3^School of Life Sciences, Shandong First Medical University & Shandong Academy of Medical Sciences, Tai’an, Shandong 271016, P.R. China

^4^Medical Science and Technology Innovation Center, Shandong First Medical University & Shandong Academy of Medical Sciences, Jinan, Shandong 250117, P.R. China

^5^These authors contributed equally

***Correspondence authors:**

Haiwei Wu; Email: [hwwu@sdfmu.edu.cn](mailto:hwwu@sdfmu.edu.cn); ORCID: 0000-0003-1813-3760

Changlong Wang; Email: [wangchanglong@sdfmu.edu.cn](mailto:wangchanglong@sdfmu.edu.cn); ORCID: 0009-0007-7534-6988

Dongsheng Zhang; Email: [ds63zhang@163.com](mailto:ds63zhang@163.com); ORCID: 0000-0003-0221-2210

**Supplemental Figures**

**Supplemental Figure1. Spatial transcriptome landscape of ST cohort. Related to Figure 1. A** Visualization of the spatial distribution of estimate scores in the ST cohort. **B** Visualization of tissue architecture using hematoxylin and eosin (H&E) images in the ST cohort.

**Supplemental Figure2. Validation of epithelial module heterogeneity and fibroblast heterogeneity. Related to Figure 2. A** Subclustering of epithelial cells, colored and labeled by subtypes. **B** Bar plots depicting the proportion of epithelial subtypes within each tumor progression stage. **C** Violin plot showing cancer plasticity module scores and CNV scores across epithelial subtypes. **D** Pseudotime trajectories for epithelial cells based on monocle, showing pseudotime (left), tumor progression stage (right). **E** Pseudotime trajectories for epithelial cells based on monocle, showing each epithelial subtype. **F** Heatmap of relative expression levels of cancer plasticity module genes along the epithelial activation trajectory. **G** BEAM heatmap plot displaying the expression patterns of cancer plasticity module genes. **H** Kaplan-Meier overall survival curves for TCGA patients stratified by epithelial subtype infiltration. **I** Heatmap illustrating the intercellular interactions among different cell types in the OSCC progression microenvironment. The shade of color reflects the frequency of mutual perturbations. **J** Heatmap illustrating the scaled expression levels of the top 10 highly expressed genes across each fibroblast subtype. **K** Kaplan-Meier overall survival curves for TCGA patients stratified by fibroblast subtype infiltration. **L** Violin plot illustrating the scores of each fibroblast signature across tumor progression stages within scRNA-seq profiles. **M** GO enrichment analysis of differentially expressed genes in the other four fibroblast subtypes. ***p* < 0.01, ****p* < 0.001, *****p* < 0.0001.

**Supplemental Figure3. Validation of PLAU’s essential role in fibroblast activation. Related to Figure 3. A** Spearman correlation analysis showing the correlations between the signature scores of fibroblast subclusters and the scores of cancer plasticity modules within scRNA-seq. **B** Spearman correlation analysis showing the correlations between COL11A1^+^ Fib signature and cancer plasticity module scores in ST cohort and the different spatial domains of AS, IF, and TC. **C** Violin plot illustrating the expression of genes (based on correlation analysis) across various stages of OSCC progression in scRNA-seq. **D** Protein-protein interaction (PPI) network in the cancer plasticity module. **E** Violin plot depicting the expression levels of the PLAU gene across various epithelial subtypes. **F** Spearman correlation analysis showing the correlations between the COL11A1^+^ Fib signature and the expression levels of PLAU in epithelial cells within scRNA-seq. **G** PLAU expression levels in normal tissue, oral leukoplakia and tumor from the GSE227919 database. **H** PLAU expression levels in normal tissue and tumor from the TCGA database (left), GSE184616 database (middle) and GSE37991 database (right). **I** Survival analysis showing the correlation between PLAU expression levels and overall survival in the TCGA (left), GSE41613 (middle), and GSE42743 (right) databases. **J** Western blot analysis and corresponding quantification of PLAU expression levels in different groups (n = 3). **K** Western blot analysis and corresponding quantification conducted to detect the protein levels of activated markers (COL11A1, FAP, α-SMA) in PAFs after treatment with varying concentrations of PLAU protein for 6 hours (n = 3). **L** Western blot analysis and corresponding quantification conducted to detect the protein levels of activated markers (COL11A1, FAP, α-SMA) in PAFs after treatment with varying concentrations of PLAU protein for 12 hours (n = 3). **M** Western blot analysis and corresponding quantification conducted to detect the protein levels of activated markers (COL11A1, FAP, α-SMA) in PAFs after treatment with varying concentrations of PLAU protein for 24 hours (n = 3). **N** Western blot analysis and corresponding quantification conducted to detect the protein levels of activated markers (COL11A1, FAP, α-SMA) in CAFs after treatment with varying concentrations of PLAU protein for 6 hours (n = 3). **O** Western blot analysis and corresponding quantification conducted to detect the protein levels of activated markers (COL11A1, FAP, α-SMA) in CAFs after treatment with varying concentrations of PLAU protein for 12 hours (n = 3). Data are presented as the mean ± SEM. ns for not significant; **p* < 0.05, ***p* < 0.01, ****p* < 0.001, *****p* < 0.0001.

**Supplemental Figure4. ITGB1 is a key molecule for fibroblast activation. Related to Figure 4. A** Dot plot illustrating the levels of cell-cell communication and the strengths of specific interactions between COL11A1^+^ Fib (source) and epithelial cell cluster C4 (target). **B** Violin plot showing the expression level of ITGB1 across various fibroblast subtypes. **C** Spearman correlation analysis revealing the relationship between PLAU expression levels and ITGB1 expression levels in the ST cohort, examining the relationships across different spatial domains of AS, IF, and TC. **D** Spearman correlation analysis revealing the relationship between PLAU and ITGB1 expression levels in fibroblasts in scRNA-seq. **E** Western blot analysis and corresponding quantification of ITGB1 expression levels in different groups (n = 3). Data are presented as the mean ± SEM. ns for not significant; ***p* < 0.01, ****p* < 0.001.

**Supplemental Figure5. Validation of TGF-β signaling pathway activation. Related to Figure 5. A** Co-IP analysis of the effect of ITGB1 knockdown on the association between TGFβRI and TGFβRII. **B** Co-IP analysis of the effects of FAK inhibitor on the levels of tyrosine phosphorylation on TGFβRI. **C** Violin plot illustrating the scores of TGF-β signaling pathway within all cell types. **D** Violin plot illustrating the scores of TGF-β signaling pathway within fibroblast subtypes.

**Supplemental Figure6. CREB3L1 is the most critical transcription factor for COL11A1^+^ Fib. Related to Figure 6. A** Heatmap depicting the area under the curve (AUC) scores for expression regulation by transcription factors within each fibroblast subtype, as estimated by SCENIC. **B** The binding motif analysis in COL11A1^+^ Fib. All the significances of the binding motifs were identified by normalized enrichment score. **C** Western blot analysis and corresponding quantification in CREB3L1 knockdown CAFs (n = 3). **D** Western blot analysis and corresponding quantification in IRF9 knockdown CAFs (n = 3). **E** Western blot analysis and corresponding quantification in STAT1 knockdown CAFs (n = 3). **F** RT-qPCR profiling of COL11A1 mRNA levels (n = 4). **G** DNA-binding motifs of CREB3L1 according to the JASPAR database. **H** Schematic of CREB3L1 binding site in the COL11A1 promoter. Data are presented as the mean ± SEM. ns for not significant; ***p* < 0.01, ****p* < 0.001, *****p* < 0.0001.

**Supplemental Figure7. Treg enriches around COL11A1^+^ Fib. Related to Figure 7.**

**A** Heatmap showing the spearman correlation coefficients between the signature scores of T cells, B cells, NK cells, and myeloid cells and COL11A1^+^ Fib across different spatial domains in ST cohort. **B** Heatmap of Spearman coefficients among Treg, ECM and COL11A1^+^ Fib signature scores across spatial domains. **C** Representative flow-cytometry staining and gating. **D** Western blot analysis and corresponding quantification in COL11A1 knockdown CAFs (n = 3). Data are presented as the mean ± SEM. ns for not significant; **p* < 0.05, ***p* < 0.01, ****p* < 0.001.

**Supplemental Figure8. Pan-cancer cohort cell clustering and spatial transcriptome landscape. Related to Figure 8.** **A** t-SNE plots of fibroblast clusters in pre-treatment and post-treatment tissues from patients with different responses in the TNBC cohort. **B** t-SNE plots of fibroblast clusters in pre-treatment and post-treatment tissues from patients with different responses in the CRC cohort. **C** Visualization of the spatial distribution of estimate scores (left) and spatial domains (right) in NSCLC cohort.
